# Combining higher accumulation of amylopectin, lysine and tryptophan in maize hybrids through genomics-assisted stacking of *waxy1* and *opaque2* genes

**DOI:** 10.1038/s41598-021-04698-3

**Published:** 2022-01-13

**Authors:** Zahirul A. Talukder, Vignesh Muthusamy, Rashmi Chhabra, Nisrita Gain, Shashidhar B. Reddappa, Subhra J. Mishra, Ravindra Kasana, Vinay Bhatt, Gulab Chand, Ashvinkumar Katral, Brijesh K. Mehta, Satish K. Guleria, Rajkumar U. Zunjare, Firoz Hossain

**Affiliations:** 1 ICAR-Indian Agricultural Research Institute (IARI), New Delhi, India; 2 ICAR-Indian Grassland and Fodder Research Institute (IGFRI), Jhansi, India; 3 CSK-Himachal Pradesh Krishi Vishvavidyalaya (CSK-HPKV), Bajaura, India

**Keywords:** Biotechnology, Genetics, Plant sciences

## Abstract

Waxy maize rich in amylopectin has emerged as a preferred food. However, waxy maize is poor in lysine and tryptophan, deficiency of which cause severe health problems. So far, no waxy hybrid with high lysine and tryptophan has been developed and commercialized. Here, we combined recessive *waxy1* (*wx1*) and *opaque2* (*o2*) genes in the parental lines of four popular hybrids (HQPM1, HQPM4, HQPM5, and HQPM7) using genomics-assisted breeding. The gene-based markers, *wx-2507F/RG* and *phi057* specific for *wx1* and *o2*, respectively were successfully used to genotype BC_1_F_1_, BC_2_F_1_ and BC_2_F_2_ populations. Background selection with > 100 SSRs resulted in recovering > 94% of the recurrent parent genome. The reconstituted hybrids showed 1.4-fold increase in amylopectin (mean: 98.84%) compared to the original hybrids (mean: 72.45%). The reconstituted hybrids also showed 14.3% and 14.6% increase in lysine (mean: 0.384%) and tryptophan (mean: 0.102%), respectively over the original hybrids (lysine: 0.336%, tryptophan: 0.089%). Reconstituted hybrids also possessed similar grain yield (mean: 6248 kg/ha) with their original versions (mean: 6111 kg/ha). The waxy hybrids with high lysine and tryptophan assume great significance in alleviating malnutrition through sustainable and cost-effective means. This is the first report of development of lysine and tryptophan rich waxy hybrids using genomics-assisted selection.

## Introduction

Maize grains are used as food, feed and industrial products worldwide^1^. Waxy maize, popularly known as ‘sticky’ maize or ‘glutinous’ maize, possesses 95–100% amylopectin compared to 70–75% in traditional maize^2,3^. Immature waxy cobs and dried grains are an essential part of the human diet in East and South-East Asian countries^4,5^. It is also used as vegetable, and various breakfast and snack items; and consumed as staple food by various ethnic groups^6^. Due to its excellent qualities of fresh harvest, waxy maize is extensively used in the frozen food processing industries^7^. Amylopectin boosts energy levels and restores muscle glycogen quickly in professional athletes^8^.

Amylopectin is a highly branched polymer with α-1,4 and α-1,6 glucosidic bonds connecting the glucose units in starch molecules^9^. The *Waxy1* (*Wx1*) gene present on long arm of chromosome-9 encodes granule-bound starch synthase-I (GBSS-I) which controls amylose synthesis in maize endosperm^10,11^. The dominant/ wild type *Wx1* gene easily converts ADP-glucose to amylose, but the recessive/ mutant *wx1* gene greatly impairs the conversion, resulting in increased amylopectin accumulation^12^. The recessive *wx1* gene is also linked to tasty and savory flavor in the kernels^13^.

The nutritional value of traditional maize including waxy type is relatively poor due to low level of essential amino acids viz., lysine (0.150–0.250%) and tryptophan (0.030–0.040%)^14^. However, specific maize genotypes having *opaque2* (*o2*) mutant gene possess much higher lysine (> 0.300%) and tryptophan (> 0.070%)^15^. Symptoms of lysine and tryptophan deficiency in children include fatigue, delayed growth, loss of appetite, depression, and anxiety^14^. Being the building block for protein synthesis, lack of lysine and tryptophan affects normal growth and development in humans, and reduces work efficiency leading to the severe socio-economic implications^16^. Furthermore, low levels of lysine and tryptophan aggravate protein energy malnutrition (PEM) that affects more than a billion people worldwide^17^. Recessive *o2* gene present on the short arm of chromosome-7 enhances lysine and tryptophan levels by nearly 2-fold^18^. The dominant *O2* gene encodes a leucine zipper (bZIP) transcription factor that activates the transcription of α-zein genes^19^. Introgression of recessive *o2* coupled with the modifier loci has resulted in the development of large array of quality protein maize (QPM) cultivars that has shown great promise in addressing the PEM^15,20^.

Waxy maize hybrids and landraces rich in amylopectin have been reported in Thailand, Vietnam, Laos, Myanmar, China, Taiwan, Philippines and Korea^4^. In India, ‘*Mimban'* a waxy landrace is cultivated in the North Eastern Himalayan region, and used as a part of important component of diet^21^. However, these waxy cultivars are poor in nutritional quality due to inadequate amount of essential amino acids like lysine and tryptophan^11^. So far, no waxy maize hybrid with high lysine and tryptophan has yet been developed and commercialized elsewhere. Here, we report the development of lysine and tryptophan rich waxy hybrids by combining *wx1* and *o2* genes through genomics-assisted breeding^3,22^. Molecular marker is a preferred option to stack multiple genes into a genetic background without the need for progeny testing thereby accelerates the breeding cycle^23^. The present investigation was therefore undertaken to (1) introgress of *wx1* gene into elite *o2*-based (QPM) inbreds using marker-assisted backcross breeding (MABB), (2) evaluate the MABB-derived inbreds and reconstituted hybrids for amylopectin, lysine and tryptophan, and (3) assess the performance of MABB-derived inbreds and reconstituted hybrids for grain yield and agronomic traits.

## Materials and methods

### Plant materials

The parental inbreds viz., HKI161, HKI163, HKI193-1, and HKI193-2 were used as recurrent parents. These parents possessed wild type *Wx1* allele and were low in amylopectin. These inbreds are the parents of four popular single cross QPM hybrids [HQPM1 (HKI193-1 × HKI163), HQPM4 (HKI193-2 × HKI161), HQPM5 (HKI163 × HKI161) and HQPM7 (HKI193-1 × HKI161)] in India. These commercial QPM hybrids have been adapted to diverse agro-ecologies of India (Table S1). A waxy inbred, MGU-102-*wx1* possessed high amylopectin (97.82%) and was used as the donor for the recessive *wx1* gene. MGU-102-*wx1* had low levels of lysine (0.245%) and tryptophan (0.043%). All the recurrent parents possessed high lysine and tryptophan due to presence of recessive *o2* gene. The donor waxy inbred had white kernels, but all of the recurrent parents were yellow in colour. Recurrent parents were crossed with donor parent, and four backcross populations viz., cross-I (HKI161 × MGU-102-*wx1*), cross-II (HKI163 × MGU-102-*wx1*), cross-III (HKI193-1 × MGU-102-*wx1*), cross-IV (HKI193-2 × MGU-102-*wx1*), were used to stack *wx1* and *o2* alleles. The detailed information of the recurrent and donor parents is given in (Table S2).

### Backcross- and self-progenies

**Figure 1:**
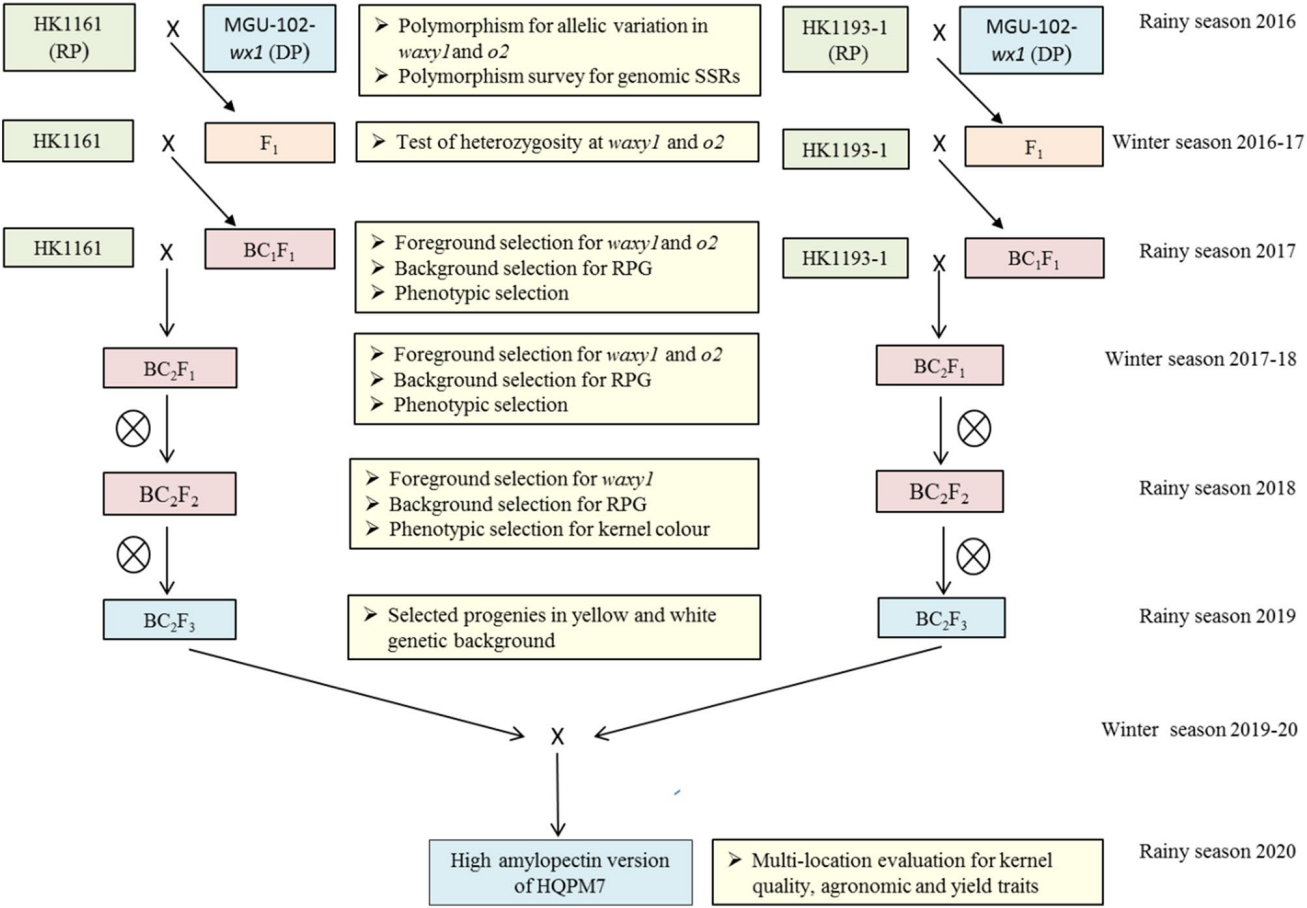
Marker-assisted backcross breeding (MABB) scheme followed for development of amylopectin rich waxy version of HQPM7. RP: recurrent parent: DP: donor parent.

The recurrent inbreds (as female) and donor inbred (as male) showing polymorphism for gene-based markers specific to both *wx1* and *o2* genes were crossed during the rainy season (July–November, 2016) at IARI, Delhi (28° 09′ N, 77° 13′ E, 229 MSL). F_1_s were grown during the winter season (December, 2016–April, 2017) at IIMR-Winter Nursery Centre (WNC), Hyderabad (17° 19′ N, 78° 25′ E, 542.6 MSL). BC_1_F_1_ progenies were grown at Delhi during the rainy season (2017), and foreground selection was carried out using the *wx1* and *o2* specific markers. The foreground positive plants along with high recovery of the recurrent parent genome (RPG), maximum phenotypic similarity to recurrent parents and endosperm opaqueness of 25–50% were backcrossed to the respective recurrent parents^15^. The BC_2_F_1_ populations raised at Hyderabad during winter season (2017–2018), and were subjected to foreground-, background- and phenotypic selection were carried out. The foreground positive plants with a maximum RPG, morphological similarity and similar kernel opaqueness (25–50%) to their recurrent parents were selfed. The BC_2_F_2_ progenies were grown during the rainy season (2018) at Delhi. Foreground positive plants homozygous for *wx1* and *o2* gene were subjected to the background- and phenotypic- selection including the kernel modification. The selected plants were self-pollinated to generate BC_2_F_3_ progenies during rainy season (2019) at Delhi (Table S3). White kernel progenies with 25–50% opaqueness in endosperm were chosen in each of the three genetic backgrounds. In all the BC_1_F_1_, BC_2_F_1_ and BC_2_F_2_ generations, kernels with 75–100% opaqueness were not considered^15^. The details of backcross- and self- generations grown at different locations and seasons are described in Table S3, while marker-assisted backcross breeding (MABB) scheme^22,23^ followed in the present study is represented in Fig. 1.

### DNA isolation and polymerase chain reaction amplification and electrophoresis

The CTAB method was used to isolate genomic DNA from young seedlings (3–4 leaf stage)^24^. Polymerase chain reaction (PCR) amplification and electrophoresis of the PCR products for the *wx1* and *o2* genes were performed using protocol standardized at Maize Genetics Unit, IARI^4,22^. PCR was performed in 20 μl volume on Veriti 96-well thermal cycler (M/s. Applied Biosystems) using GeneDirex OnePCR reaction mixture. Amplification of PCR products was performed with a ‘touch-down 60’ procedure as per Duo et al.^1^ Electrophoretic separation of the PCR products was performed using 4% agarose (Lonza, Rockland, ME USA) at 100–120 V for 3–4 h with a 50 bp DNA ladder (MBA-Fermentas). Photographs of the amplified products was captured using gel documentation system (AlphaInnotech, California, USA).

## Marker-assisted foreground selection for *wx1* and *o2* gene

Hybridity testing was undertaken in F_1_s using markers specific to *wx1* and *o2* genes. Foreground selection was performed in BC_1_F_1_, BC_2_F_1_, and BC_2_F_2_ generations. Gene based *InDel* marker, *wx-2507F/RG* was used for selection of *wx1* gene^25^. Heterozygous plants (*Wx1*/*wx1*) were selected in the BC_1_F_1_ and BC_2_F_1_, while homozygotes (*wx1*/*wx1*) were selected in BC_2_F_2_. SSR, *phi057* was used to genotype the populations and homozygotes (*o2o2*) were selected in BC_1_F_1_^26^. The details information of markers used in foreground selection are presented in Table S4.

## Marker-assisted background selection for recurrent parent genome

A set of > 320 genome-wide SSRs covering all the 10 chromosomes of the maize genome were used for identifying polymorphic markers between the respective recurrent and donor parents (Table 1). The sequence of SSR primers was retrieved from the maize genome database (www.maizegdb.org) and was custom synthesized (Sigma Tech., USA). PCR amplification and scoring of amplicons of SSRs employed in background selection were carried out as per Hossain et al.^22^ Polymorphic SSRs between the recurrent and donor parents were used to recover the RPG in individuals from the BC_1_F_1_, BC_2_F_1_ and BC_2_F_2_ populations.

**Table 1:** Percent polymorphism and distribution of SSRs used in background selection.

LG	No. of SSRs screened	HKI161 × MGU-102-*wx1*		HKI163 × MGU-102-*wx1*		HKI93-1 × MGU-102-*wx1*		HKI93-2 × MGU-102-*wx1*	
		NP	Pol (%)	NP	Pol (%)	NP	Pol (%)	NP	Pol (%)
1	26	11	42.31	10	38.46	10	38.46	11	42.31
2	29	12	41.38	11	37.93	10	34.48	10	34.48
3	35	11	31.43	10	28.57	12	34.29	13	37.14
4	36	16	44.44	14	38.89	12	33.33	11	30.56
5	38	11	28.95	11	28.95	11	28.95	8	21.05
6	32	11	34.38	12	37.50	10	31.25	9	28.13
7	34	10	29.41	9	26.47	11	32.35	12	35.29
8	36	10	27.78	11	30.56	9	25.00	11	30.56
9	28	10	35.71	10	35.71	8	28.57	9	32.14
10	26	10	38.46	9	34.62	9	34.62	11	42.31
Total	320	112	35.00	107	33.44	102	31.86	105	32.81

*LG* Linkage group, *NP* No. of observed polymorphic markers, *Pol.* (%), Polymorphism percentage, *SSR* Simple Sequence Repeats

### Agronomic evaluation of MABB-derived inbreds

MABB-derived inbreds (three from each of the four genetic background) and their recurrent parents were evaluated in randomized complete block design (RCBD) with two replications at the IARI, Delhi during the rainy season (2020). Each inbred was grown in a 3 m row, with a 75 cm row-to-row and 20 cm plant-to-plant distance. Inbreds were characterized for five important agronomic traits [days to 50% anthesis (MF), days to 50% silking (FF), plant height (PH), ear height (EH) and grain yield (GY)] and 31 morphological characters pertaining to distinctness, uniformity and stability (DUS)^27^. Standard agronomic practices were followed to raise the good crop. Two to three plants per entry were self-pollinated to avoid any xenia effects caused by foreign pollens, and the selfed grains were analyzed for amylopectin, lysine and tryptophan. Characters namely MF, FF, PH, EH and GY were recorded from open pollinated plants.

### Agronomic evaluation of reconstituted hybrids

Selected three BC_2_F_3_ progenies from each of the four inbreds were used to reconstitute 12 F_1_ hybrids during the winter season (2019–20) at Hyderabad. Three versions of the reconstituted hybrids (-A, -B, and -C) and their corresponding original hybrid in each of the four hybrid combinations were evaluated in RCBD with two replications at three diverse maize growing zones of the country namely (1) IARI, Delhi, (2) CSK-HPKV, Bajaura (31° 85′ N, 77° 16′ E, 1090 m MSL) and (3) IGFRI, Jhansi (25° 26′ N, 78° 30′ E, 216 m MSL) during rainy season (2020). Standard agronomic practices were adopted for raising the hybrids. The hybrids were evaluated using 3 m row with a plant-to-plant and row-to-row distance 20 cm and 75 cm, respectively. Two to three plants in each hybrid were self-pollinated to avoid xenia effects. Selfed-seeds was used for the estimation of amylopectin, lysine and tryptophan. Morphological characteristics such as MF, FF, PH, EH, and GY were recorded from open-pollinated plants. The hybrids were also characterized for 31 DUS characters^27^.

### Analysis of amylopectin

Self-pollinated grains were used to estimate amylopectin from maize kernels. Absolute amylose content was estimated as per Gibbon et al.^28^ with minor modifications. Around 8–10 dried maize seeds were ground into seed powder with a diameter of < 0.2 mm using seed grinder (Cyclotec Sample Mill-1093, Sweden). Weighted 100 mg of seed powder was treated with 500 µl of 80% ethanol and vortexed for a short time. The sample tubes were centrifuged for 5 min at 10,000 rpm and supernatant was separated. The residues of the samples were again treated with 10% toluene and centrifuged for 5 min at 10,000 rpm and supernatant was separated. The process was repeated until the supernatant was clear of white layer. The supernatant was discarded, and the residue was fully dried in an incubator at 80 °C for 3–4 h. The resulting residue represented starch with a < 5% impurity level. 25 mg of the starch residue was placed into a 50 ml falconer tube. It was solubilized with 2.5 ml 1 M NaOH and mixed properly, and heated for 20 min in a hot water bath at 80 °C. The volume was adjusted to 25 ml with double distilled water after the samples were cooled to room temperature. 1.25 ml samples were transferred from the above sample into a new 50 ml falconer tube and treated with 125 µl 1 N acetic acid, 100 µl 1 M NaOH, and 500 µl of I_2_-KI solution. The samples were incubated at room temperature for 20 min to generate colour, then measured at 620 nm for absorbance (G-Biosciences Spectrophotometer, BT-UVS-SBA-E, BenchTop). The percent of amylose was calculated using the average of three technical replicates. The percent amylopectin was obtained by subtracting amylose from 100.

### Analysis of lysine, and tryptophan

The lysine and tryptophan of maize kernels was estimated using UHPLC (Dionex Ultimate 3000 System, Thermo Scientific, Massachusetts, USA). The selfed seeds were dried and ground into powder, and further used for estimation of lysine and tryptophan^29^. The flour of the grains was acid hydrolyzed using 800 μl of 6 N HCl, 100 μl of 0.1 N HCl, 100 μl of nor-leucine and 10 μl of phenol for 16 h at 110 °C. Two mobile phases, A and B consisted of buffer and organic phase in the ratio of 9:1 (v/v) and 1:9 (v/v), respectively were used for estimation of lysine. Buffer phase for lysine contained tetra-methyl ammonium chloride and sodium acetate trihydrate (pH 3.5), while organic phase had acetonitrile and methanol (49:1, v/v). In case of tryptophan, alkaline hydrolysis (2 ml of 4 M NaOH and 200 μl of 0.1% ascorbic acid for 16 h at 110 °C) was performed. The mobile phase for tryptophan consisted of water and acetonitrile in the ratio of 95:5. The samples were injected separately in UHPLC through Acclaim 120 C_18_ column (5 μm, 120 Å, 4.6 × 150 mm) with a flow rate of 1.0 and 0.7 ml/min, and detected using RS 3000 photodiode array (PDA) detector at 265 and 280 nm, respectively. The concentration of lysine and tryptophan was estimated in three technical replicates by standard regression curve derived using dilutions of external standards (AAS 18-5ML, Sigma Aldrich).

### Statistical analysis

Chi-square analysis was used to test the goodness of fit of the observed segregation pattern of *wx1* across segregating populations (BC_1_F_1_, BC_2_F_1_ and BC_2_F_2_), as well as *o2* in the BC_1_F_1_ generation^22^. The amplicons of SSRs used in background selection were scored as “A” for the recurrent parent, “B” for the donor parent, and “H” for the heterozygous genotype. Recovery of RPG was estimated using formula^30^, RPG (%) = [A + (0.5H)/ (A + B + H)] × 100. Graphical Geno Types (GGT) version 3.0 was also used to determine the recovery of RPG in selected backcross-derived progenies^31^. Graphical representations on amylopectin, lysine and tryptophan in each genotype were made using Microsoft Excel (2013). Windostat v10 software was used to analyze the agronomic and biochemical data.

### Research involving plants

No approvals were required for the study, which complied with all relevant regulations.

## Results

### Marker polymorphism among parents

**Figure 2:**
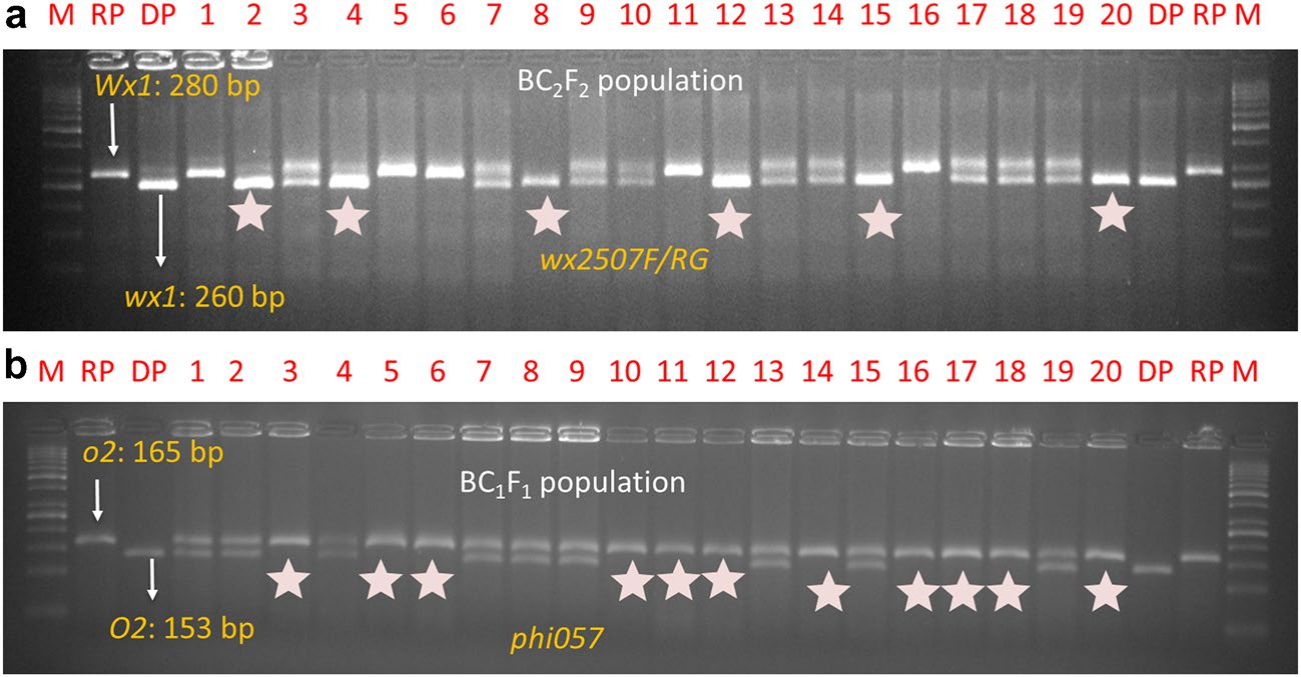
(**A**) Foreground selection for *wx1* gene in BC_2_F_2_ generation. DP: donor parent, RP: recurrent parent, M: Ladder 50 bp, Star indicates homozygotes recessive, (**B**) Foreground selection for o2 gene in BC_1_F_1_ generation. DP: donor parent, RP: recurrent parent, M: Ladder 50 bp, Star indicates homozygotes recessive.

Gene-based *InDel* marker, *wx-2507F/RG* was polymorphic between recurrent (HKI161, HKI163, HKI193-1 and HKI193-2) and donor (MGU-102-*wx1*) parents. *wx-2507F/RG* amplified 280 bp fragment in all the four recurrent inbreds, while it amplified 260 bp fragment in waxy donor line (Fig. 2A). Gene-based SSR, *phi057* produced 165 bp allele in all four recurrent parents, while the donor generated 153 bp allele (Fig. 2B). A range of 102–273 plants across BC_1_F_1_, BC_2_F_1_ and BC_2_F_2_ were subjected to foreground selection using *wx1* and *o2* gene (Table 2, Fig. 2). A total of 112, 107, 102 and 105 polymorphic SSRs with polymorphism of 35.00%, 33.44%, 31.86% and 32.81% were observed in HKII61 × MGU-102-*wx1*, HKI163 × MGU-102-*wx1*, HKI193-1 × MGU-102-*wx1* and HKI193-2 × MGU-102-*wx1*, respectively (Table 1). The number of polymorphic markers per chromosome ranged from 8 to 16 across crosses.

**Table 2:** Segregation pattern of *wx1* and *o2* in different backcrosses and self-generations.

S. no.	Cross	Generation	N	*Wx1/Wx1*	*Wx1/wx1*	*wx1/wx1*	χ^2^	P-value	N	*O2/o2*	*o2/o2*	χ^2^	*P*-value
1	HKI161 × MGU-102-*wx1*	BC_1_F_1_	115	64	51	-	1.47	0.23 ^NS^	51	18	33	4.52	0.03*
2		BC_2_F_1_	108	49	59	-	0.93	0.34 ^NS^	59	–	72	–	–
3		BC_2_F_2_	198	43	105	50	1.22	0.54 ^NS^	50	–	50	–	–
4	HKI163 × MGU-102-*wx1*	BC_1_F_1_	105	59	46	-	1.61	0.21 ^NS^	46	20	26	1.04	0.31 ^NS^
5		BC_2_F_1_	110	62	48	-	1.78	0.18 ^NS^	48	–	48	–	–
6		BC_2_F_2_	273	74	139	60	1.53	0.47 ^NS^	60	–	60	–	–
7	HKI193-1 × MGU-102-*wx1*	BC_1_F_1_	104	57	47	-	0.96	0.32 ^NS^	47	27	20	1.16	0.28 ^NS^
8		BC_2_F_1_	114	60	54	-	0.32	0.57 ^NS^	54	–	54	–	–
9		BC_2_F_2_	207	50	99	58	1.01	0.60 ^NS^	58	–	58	–	–
10	HKI193-2 × MGU-102-*wx1*	BC_1_F_1_	110	48	62	-	1.78	0.18 ^NS^	62	25	37	5.76	0.02*
11		BC_2_F_1_	102	56	46	-	0.98	0.32 ^NS^	46	–	46	–	–
12		BC_2_F_2_	195	45	99	51	0.42	0.81 ^NS^	51	–	51	–	–

*Significant at *P* = 0.05.

*ns* non-significant, *N* No. of plants genotyped, *df* degrees of freedom, *Wx1,* dominant allele; *wx1,* recessive allele; *O2*, dominant allele; *o2*, recessive allele.

### Genomics-assisted selection

#### *F*_*1*_* generation*

The corresponding polymorphic markers for *wx1* and *o2* showed hybridity among all the F_1_s. as all plants revealed 280/260 bp (*Wx1wx1*) and 165/153 bp (*O2o2*) amplicons.

#### BC_1_F_1_ generation

Foreground selection using *wx1* gene in BC_1_F_1_ identified 51, 46, 47 and 62 heterozygous plants (*Wx1wx1*) in HKII61 × MGU-102-*wx1*, HKI163 × MGU-102-*wx1,* HKI193-1 × MGU-102-*wx1* and HKI193-2 × MGU-102-*wx1* populations, respectively (Table 2). The chi-square test revealed Mendelian segregation ratio of 1:1 for the *wx1* gene in all four crosses **(**Table 2). These identified heterozygous plants (*Wx1wx1*) were further subjected to foreground selection using *o2* gene. The PCR assay identified 33 homozygous plants (*o2o2*) in HKII61 × MGU-102-*wx1*, while it was 26, 20 and 37 plants in HKI163 × MGU-102-*wx1,* HKI193-1 × MGU-102-*wx1* and HKI193-2 × MGU-102-*wx1* populations, respectively. Significant segregation distortion of *o2* gene was observed in two crosses (HKII61 × MGU-102-*wx1* and HKI193-2 × MGU-102-*wx1*), while rest two crosses (HKI163 × MGU-102-*wx1*and HKI193-1 × MGU-102-*wx1*) showed 1:1 ratio (Table 2). Consequently, foreground positive plants with 25–50% opaqueness were studied for background selection using polymorphic markers. Foreground positive plants with 75–100% opaqueness were rejected. Two plants each in HKI161- (82.1% and 83.5% RPG), HKI163- (81.3% and 80.4% RPG), and HKI193-2- (82.4% and 83.3% RPG), while three plants in HKI193-1- (82.4%, 82.8% and 81.4% RPG) based populations were selected for further advancement (Table 3). In BC1F1 generation, the recovery of RPG among the selected individuals varied from 81.3 to 83.5% with an average of 82.2%.

**Table 3:** Recovery of recurrent parent genome (RPG) among introgressed progenies.

S. no.	Cross	Generation	Genotypes	RPG (%) in selected progeny	Range of RPG (%) among all progenies
1	HKI161 × MGU-102-*wx1*	BC_1_F_1_	HKI161-99	82.1	75.9–83.5
2			HKI161-107	83.5	
3		BC_2_F_1_	HKI161-99-30	92.9	89.3–93.3
4			HKI161-107-42	93.3	
5		BC_2_F_2_	HKI161-99-30-43	95.1	91.6–96.4
6			HKI161-107-42-1	95.5	
7			HKI161-107-42-10	96.4	
8	HKI163 × MGU-102-*wx1*	BC_1_F_1_	HKI163-9	81.3	73.8–81.3
9			HKI163-19	80.4	
10		BC_2_F_1_	HKI163-9-2	91.6	87.9–91.6
11			HKI163-9-35	90.7	
12			HKI163-19-3	91.1	
13		BC_2_F_2_	HKI163-9-2-13	94.4	90.7–94.4
14			HKI163-9-35-88	93.5	
15			HKI163-19-3-107	93.9	
16	HKI193-1 × MGU-102-*wx1*	BC_1_F_1_	HKI193-1-3	82.4	76.5–82.8
17			HKI193-1-6	82.8	
18			HKI193-1-14	81.4	
19		BC_2_F_1_	HKI193-1-6-55	92.2	88.7–92.2
20			HKI193-1-14-1	91.7	
21		BC_2_F_2_	HKI193-1-6-55-9	94.1	91.7–95.1
22			HKI193-1-6-55-116	95.1	
23			HKI193-1-14-1-57	94.6	
24	HKI193-2 × MGU-102-*wx1*	BC_1_F_1_	HKI193-2-4	82.4	75.1–83.3
25			HKI193-2-6	83.3	
26		BC_2_F_1_	HKI193-2-4-39	91.9	89.5–92.4
27			HKI193-2-4-20	92.4	
28		BC_2_F_2_	HKI193-2-4-39-45	94.3	91.9–95.2
29			HKI193-2-4-20-56	94.8	
30			HKI193-2-4-20-111	95.2	

#### BC_2_F_1_ generation

A total of 59 heterozygous plants (*Wx1wx1*) was identified in HKI161 × MGU-102-*wx1*, while the same was 48 in HKI163 × MGU-102-*wx1,* 54 in HKI193-1 × MGU-102-*wx1* and 46 in HKI193-2 × MGU-102-*wx1* (Table 2). In all four crosses, Mendelian inheritance ratio of 1:1 was observed for the *wx1* gene. All the heterozygous plants across population also showed homozygosity for *o2* gene. Background selection among *Wx1wx1/o2o2* plants (with 25–50% opaqueness) using polymorphic SSRs led to the recovery of 89.3–93.3% RPG in HKI161 × MGU-102-*wx1*, 87.9–91.6% in HKI163 × MGU-102-*wx1*, 88.7–92.2% in HKI193-1 × MGU-102-*wx1* and 89.5–92.4% in HKI193-2 × MGU-102-*wx1*. Two plants in each of HKI161- (92.9% and 93.3% RPG), HKI193-1- (92.2% and 91.7% RPG), and HKI193-2- (91.9% and 92.4% RPG), while three plants in HKI163- (91.6%, 90.7% and 91.1% RPG) based populations were advanced (Table 3). Across BC_2_F_1_ generations, the average recovery of RPG was 92.0% with a range from 90.7 to 93.3%.

#### BC_2_F_2_ generation

**Figure 3:**
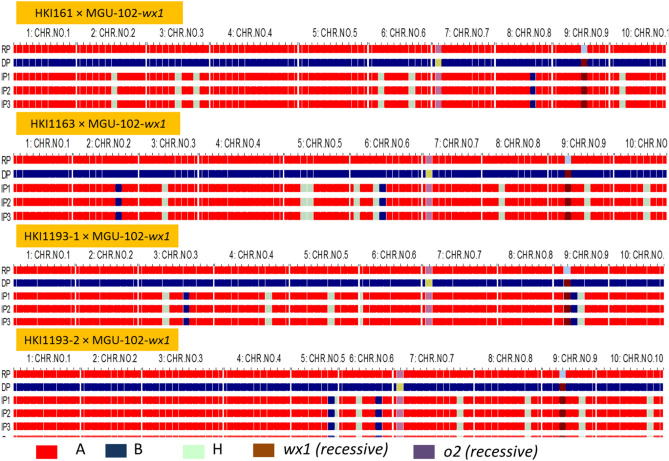
Graphical genotype of intogressed progenies across the three crosses. RP: recurrent parent; DP: donor parent; IP: introgressed progeny; CHR: Chromosome.

Foreground selection identified 50 homozygous plants (*wx1wx1*) in HKI161 × MGU-102-*wx1*, while it was 60, 58 and 51 in HKI163 × MGU-102-*wx,* HKI193-1 × MGU-102-*wx1* and HKI193-2 × MGU-102-*wx1*, respectively (Table 2). With regard to the *wx1* gene, all four crosses followed the Mendelian segregation pattern of 1:2:1. (Table 2). All the homozygous plants (*wx1wx1*) also revealed the presence of *o2* gene in homozygous condition. Screening of double-homozygous plants (*wx1wx1*/*o2o2*) having 25–50% opaqueness with background markers led to high recovery of RPG in HKI161 × MGU-102-*wx1* (91.6–96.4%), HKI163 × MGU-102-*wx1* (90.7–94.4%), HKI193-1 × MGU-102-*wx1* (91.7–95.1%) and HKI193-2 × MGU-102-*wx1* (91.9–95.2%) (Table 3). Three plants each in HKI161- (95.1%, 95.5% and 96.4%), HKI163- (94.4%, 93.5% and 93.9% RPG), HKI193-1- (94.1%, 95.1% and 94.6% RPG) and HKI193-2- (94.3%, 94.8% and 95.2% RPG) based populations were selected for further advancement (Table 3, Fig. 3). Recovery among the selected progenies ranged from 93.5 to 96.4%, with an average of 95.2%.

### Selection of BC_2_F_3_ progenies for kernel colour

BC_2_F_3_ seeds borne on BC_2_F_2_ plants with *wx1wx1*/*o2o2* genotype were selected for white colour in kernels. Seeds with yellow kernel colour were not considered for the present study. The white seeds homozygous for both *wx1* and *o2* genes were planted in order to generate BC_2_F_3_ progenies. Three progenies each in HKI161-, HKI1163-, HKI193-1- and HKI193-2- based populations were finally selected for evaluation and reconstitution of hybrids **(**Table 4).

**Table 4:** Nutritional quality attributes among introgressed progenies and their respective recurrent.

S. no.	Genotypes	Amylopectin (%)	Lysine (%)	Tryptophan (%)
1	HKI161	73.76	0.315	0.079
2	HKI161-99-30-43-290	98.94	0.364	0.093
3	HKI161-107-42-1-291	99.00	0.367	0.089
4	HKI161-107-42-10-293	98.57	0.351	0.091
5	HKI163	72.00	0.338	0.084
6	HKI163-9-2-13-302	98.24	0.378	0.094
7	HKI163-9-35-88-303	97.68	0.365	0.093
8	HKI163-19-3-107-304	98.46	0.370	0.091
9	HKI193-1	74.10	0.320	0.078
10	HKI193-1-6-55-9-317	99.31	0.391	0.092
11	HKI193-1-6-55-116-319	98.70	0.372	0.092
12	HKI193-1-14-1-57-320	99.06	0.381	0.095
13	HKI193-2	75.17	0.298	0.074
14	HKI193-2-4-39-45-321	99.23	0.351	0.087
15	HKI193-2-4-20-56-322	98.72	0.345	0.090
16	HKI193-2-4-20-111-325	98.52	0.364	0.088
CD (5%)		8.67	0.040	0.005

### Evaluation of introgressed inbreds for amylopectin

**Figure 4:**
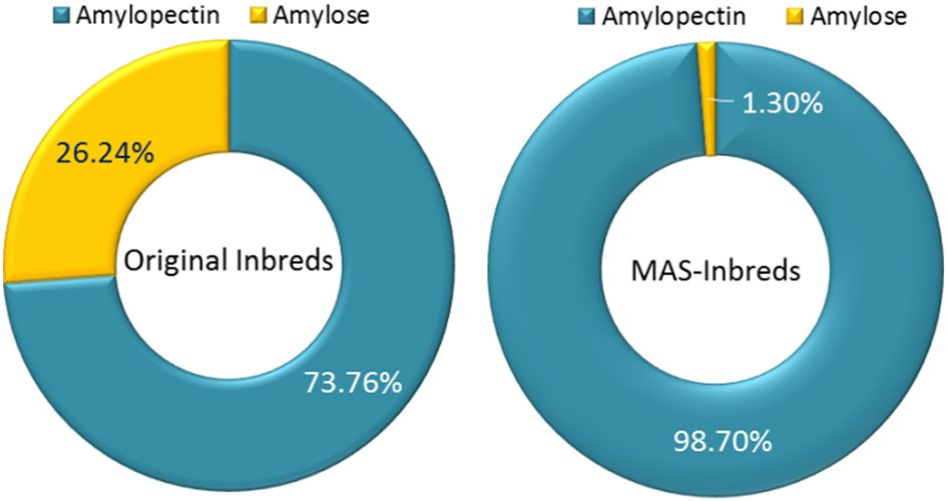
Average amylopectin and amylose content in original and MAS-derived inbreds.

Amylopectin among MABB-derived progenies of HKI161, HKI163, HKI193-1 and HKI193-2 showed substantial increase (mean: 98.70%, range: 97.68–99.31%) over their respective recurrent parents (mean: 73.76%, range: 72.00–75.17%) (Table 4, Fig. 4). All the introgressed inbreds were statistically superior to their respective recurrent parents for amylopectin content. HKI161 had 73.76% amylopectin, while its waxy versions possessed 98.94% (HKI161-99-30-43-290), 99.00% (HKI161-107-42-1-291) and 98.57% (HKI161-107-42-10-293) amylopectin. HKI163 possessed 72.00% amylopectin, and its MABB versions had 98.24% (HKI163-9-2-13-302), 97.68% (HKI163-9-35-88-303) and 98.46% (HKI163-19-3-107-304) amylopectin. Waxy versions of HKI193-1 had 99.31% [HKI193-1-6-55-9-317)], 98.70% [HKI193-1-6-55-116-319], and 99.06% [HKI193-1-14-1-57-320] amylopectin, compared to 74.10% in HKI193-1 (Table 4, Fig. 3S). HKI193-2 had 75.17% amylopectin, and its MABB versions possessed 99.23% [HKI193-2-4-39-45-321], 98.72% [HKI193-2-4-20-56-322] and 98.52% [HKI193-2-4-20-111-325] amylopectin. Overall, an average of ~ 1.4-fold increase in amylopectin was recorded among introgressed progenies. However, starch content among introgressed- (mean: 69.42%) and original- inbreds (mean: 68.05%) were statistically *at par* (Table S5).

### Evaluation of introgressed inbreds for lysine and tryptophan

**Figure 5:**
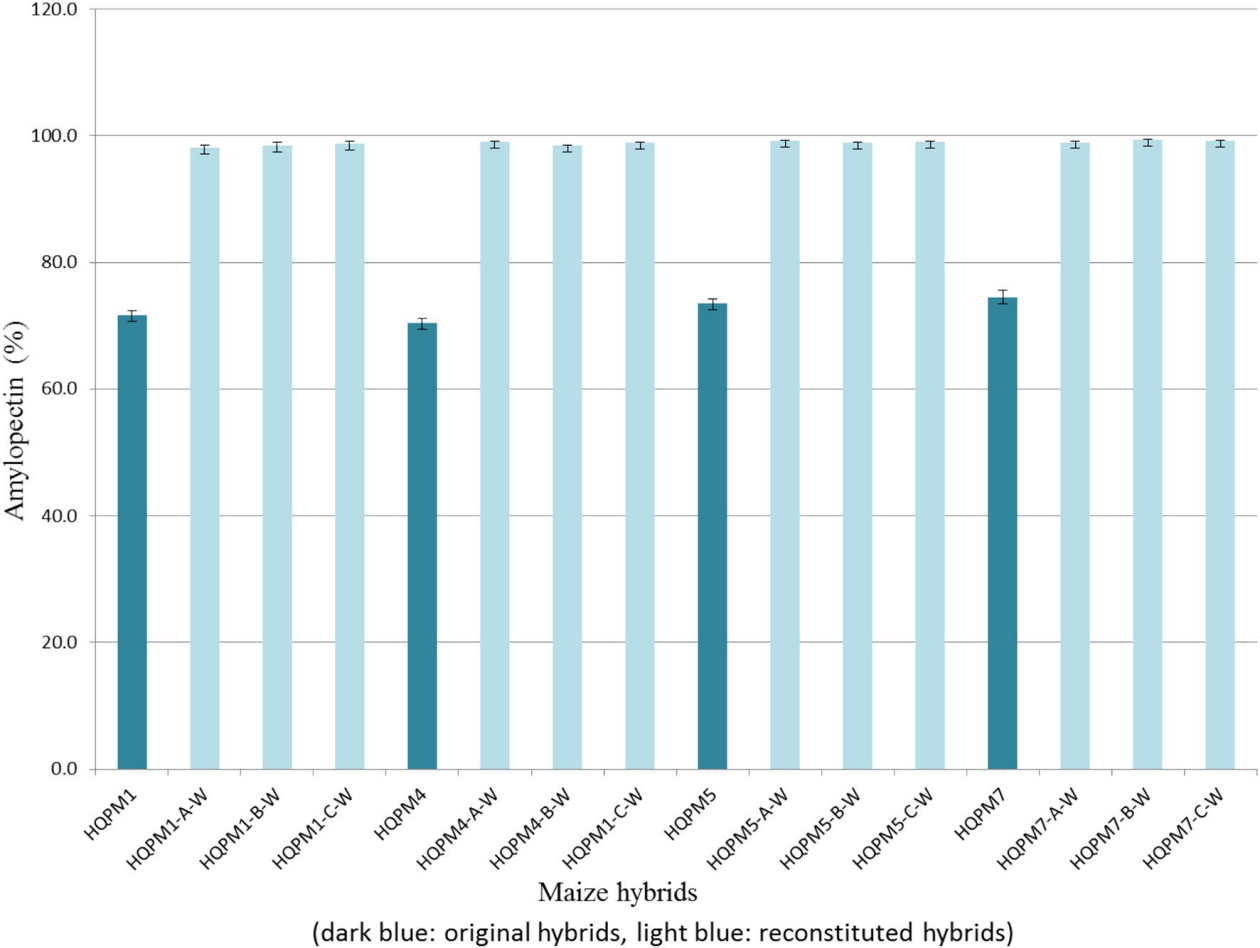
Amylopectin content in original- and reconstituted waxy-hybrids.

MABB-derived progenies of HKI161, HKI163, HKI193-1 and HKI193-2 showed higher lysine (mean: 0.367%) and tryptophan (mean; 0.091%) over their respective recurrent parents (lysine: 0.318%, tryptophan: 0.079%) (Table 4, Figs. 4S, 5S). Each of the introgressed progenies had significantly higher lysine and tryptophan over their respective recurrent parents except HKI163-9-35-88-303 and HKI163-19-3-107-304 which had statistically similar lysine with HKI163. HKI161 had 0.315% lysine and 0.079% tryptophan, while its waxy versions viz*.*, HKI161-99-30-43-290 (lysine: 0.364%, tryptophan: 0.093%), HKI161-107-42-1-291 (lysine: 0.367%, tryptophan: 0.089%) and HKI161-107-42-10-293 (lysine: 0.351%, tryptophan: 0.091%) had higher accumulation. Waxy versions viz., HKI163-9-2-13-302 (lysine: 0.378%, tryptophan: 0.094%), HKI163-9-35-88-303 (lysine: 0.365%, tryptophan: 0.093%) and HKI163-19-3-107-304 (lysine: 0.370%, tryptophan: 0.091%) possessed better nutritional quality compared to HKI163 (lysine: 0.338%, tryptophan: 0.084%). In case of HKI193-1, lysine and tryptophan was 0.320% and 0.078%, respectively, while waxy versions viz., HKI193-1-6-55-9-317 (lysine: 0.391%, and tryptophan: 0.092%), HKI193-1-6-55-116-319 (lysine: 0.372%, tryptophan: 0.092%), and HKI193-1-14-1-57-320 (lysine: 0.381%, tryptophan: 0.095%) possessed higher accumulation. MABB-versions viz., HKI193-2-4-39-45-321 (lysine: 0.351%, tryptophan: 0.087%), HKI193-2-4-20-56-322 (lysine: 0.345%, tryptophan: 0.09%) and HKI193-2-4-20-111-325 (lysine: 0.364%, tryptophan: 0.088%) also possessed superior nutritional quality over original inbred, HKI193-2 (lysine: 0.298%, tryptophan: 0.074%) (Table 4). Overall, introgressed progenies possessed 1.2-fold more lysine and tryptophan over the original inbreds.

### Evaluation of introgressed inbreds for yield and morphological characters

In general, introgressed progenies and their respective recurrent parents showed statistically similar levels of grain yield, days to anthesis, days to silking, plant height and ear height (Table 5). The grain yield of HKI161 was 1876 kg/ha, while the same in waxy versions ranged from 1742 to 2142 kg/ha (mean: 1991 kg/ha). HKI163 had grain yield of 2080 kg/ha, while its *wx1* introgressed lines produced 1951–2107 kg/ha (mean: 2016 kg/ha) of grain yield. HKI193-1 and HKI193-2 produced grain yield of 1698 kg/ha and 1876 kg/ha, while their introgressed lines had grain yield of 1653–1920 kg/ha (mean: 1754 kg/ha) and 1653–2053 kg/ha (mean: 1949 kg/ha), respectively. However, significant difference was observed in few cases viz*.*, (1) grain yield (HKI161 and HKI161-107–42-1–291, HKI193-1 and HKI193-2-4-20-111-325, and (2) days to anthesis and silking (HKI193-1 and HKI193-1-6-55-9-317, HKI193-1-6-55-116-319 and HKI193-1-14-1-57-320) (Table 5). The waxy inbreds also showed a high degree of phenotypic similarity for DUS characters with their recurrent parents (Table S7). However, the marker-assisted selection (MAS)-derived inbreds differed from their original inbreds for few DUS characteristics as well. For example, anthocyanin colouration of brace root was present in HKI161, while it was absent in HKI161-99-30-43-290, HKI161-107-42-1-291 and HKI161-107-42-10-293 (Table S7a). Similarly, anthocyanin colouration of brace root was absent in HKI193-1, while it was found present in all the three versions (Table S7c).

**Table 5:** Morphological characterization of introgressed progenies and their respective recurrent.

S. no.	Inbreds	GY (kg/ha)	MF (days)	FF (days)	PH (cm)	EH (cm)
1	HKI161	1876	50.5	53.5	114.5	55.2
2	HKI161-99-30-43-290	1742	48.5	51.5	108.5	56.0
3	HKI161-107-42-1-291	2142	51.5	54.5	113.3	51.3
4	HKI161-107-42-10-293	2089	50.0	53.0	116.7	60.0
5	HKI163	2080	54.0	57.0	125.2	75.2
6	HKI163-9-2-13-302	2107	51.5	54.5	123.8	71.7
7	HKI163-9-35-88-303	1951	54.5	57.5	127.8	71.7
8	HKI163-19-3-107-304	1991	54.5	57.5	126.8	77.2
9	HKI193-1	1698	54.0	57.0	114.2	47.2
10	(HKI193-1)-6-55-9-317	1920	50.0	53.0	104.3	48.5
11	(HKI193-1)-6-55-116-319	1653	50.5	53.5	116.5	51.8
12	(HKI193-1)-14-1-57-320	1689	50.0	53.0	109.3	57.0
13	HKI193-2	1876	52.0	55.0	117.2	61.7
14	(HKI193-2)-4-39-45-321	1653	54.0	56.5	115.3	60.7
15	(HKI193-2)-4-20-56-322	2053	54.0	57.0	122.8	59.2
16	(HKI193-2)-4-20-111-325	2142	52.5	55.5	119.0	60.2
CD (5%)		251.60	2.91	2.88	14.25	9.25

*GY* Grain yield, *MF* days to anthesis, *FF* days to silking, *PH* plant height, *EH* ear height, *CD *Critical difference

### Evaluation of MAS-derived hybrids for amylopectin

**Figure 6:**
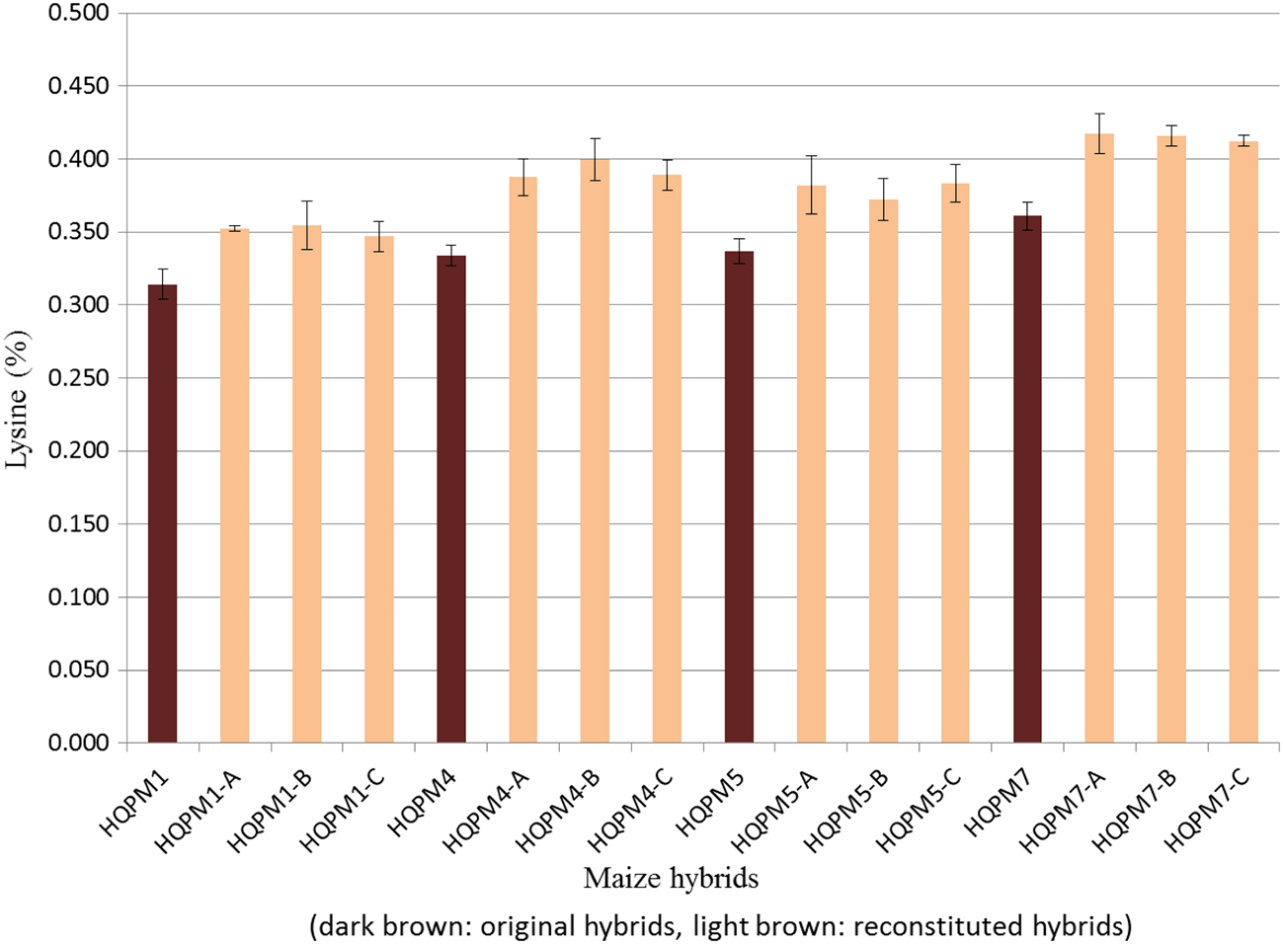
Lysine concentration in original- and reconstituted waxy-hybrids.

The amylopectin of the reconstituted hybrids increased significantly from 72.45% in the original hybrids to 98.84% in the MABB-derived hybrids across three locations **(**Table S6, Fig. 6S). The newly derived waxy hybrids possessed amylopectin ranging from 98.07 to 99.37% compared to 70.43–74.36% among the original hybrids (Fig. 5). All the reconstituted hybrids showed statistically higher amount of amylopectin from their original hybrids. The original HQPM1 possessed 71.60% amylopectin, whereas its reconstituted hybrids had 98.07% (HQPM1-A), 98.48% (HQPM1-B), and 98.78% (HQPM1-C) amylopectin. HQPM4 had 70.43% amylopectin, while waxy versions of the hybrids possessed 99.04% (HQPM4-A), 98.42% (HQPM4-A) and 98.81 (HQPM4-A) amylopectin. On the other hand, reconstituted hybrids had 99.14% (HQPM5-A), 98.85% (HQPM5-B), and 99.00% (HQPM5-C) amylopectin, compared to 73.44% in HQPM5. Similarly, amylopectin of HQPM7 was 74.36%, and its waxy versions had 98.95% (HQPM7-A), 99.37% (HQPM7-B), and 99.11% (HQPM7-C) amylopectin. Amylopectin levels in the reconstituted waxy hybrids was increased by 1.4-fold over original versions across locations. However, starch content of the original (mean: 70.20%) and reconstituted (mean: 71.66%) versions of the hybrids were statistically *at par* (Table S5).

### Evaluation of MAS-derived hybrids for lysine and tryptophan

**Figure 7:**
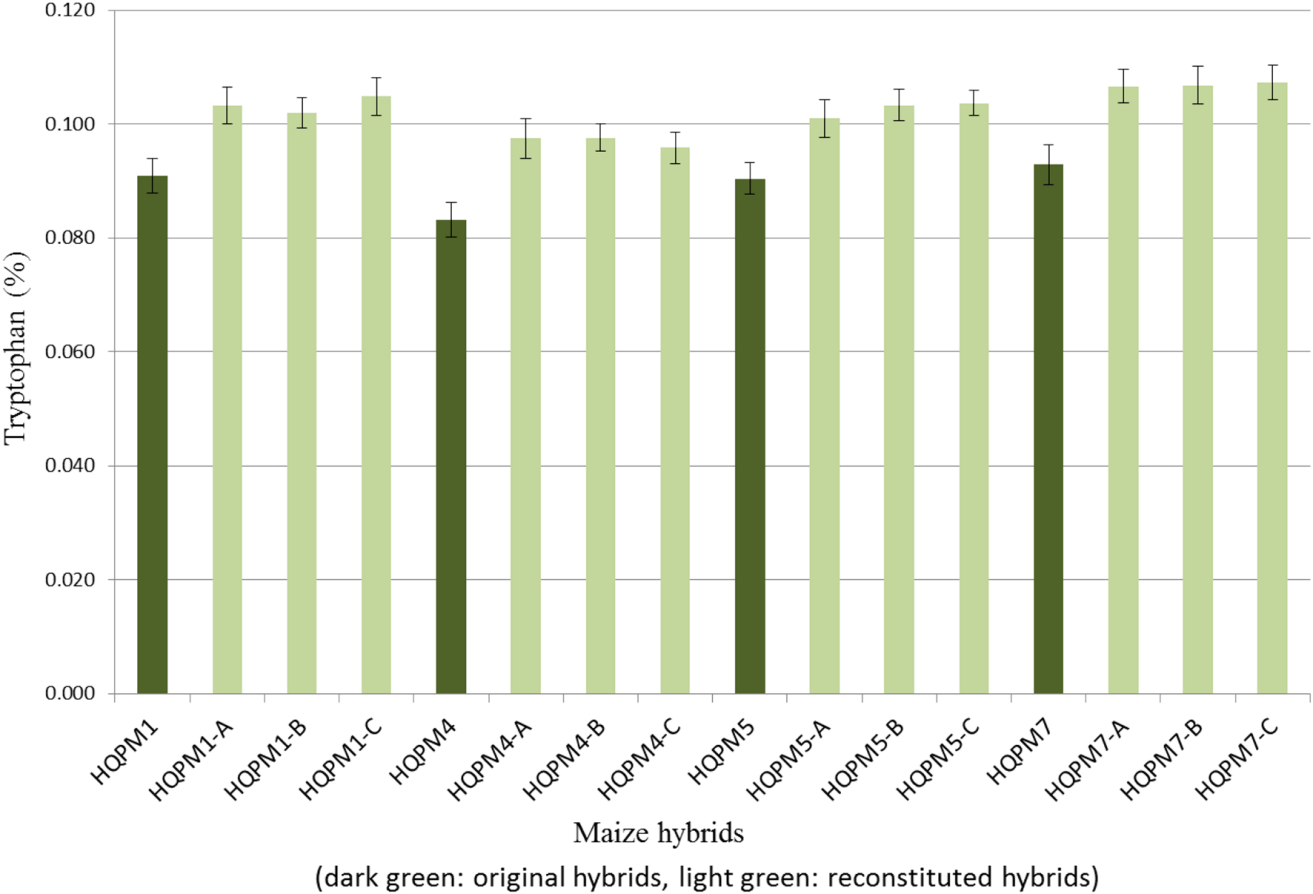
Tryptophan concentration in original- and reconstituted waxy-hybrids.

The newly derived waxy hybrids had significantly higher lysine (mean: 0.384%, range: 0.347–0.417%) and tryptophan (mean: 0.102%, range: 0.096–0.107%) compared to lysine (mean: 0.336%, range: 0.314–0.361%) and tryptophan (mean: 0.089%, range: 0.083–0.093%) in the original hybrids (Figs. 6, 7). All the reconstituted hybrids possessed statistically higher amount of lysine and tryptophan over the original versions (Table S6). The lysine and tryptophan in HQPM1 were 0.314% and 0.091%, while waxy HQPM1 version of the reconstituted hybrids viz., HQPM1-A (lysine: 0.353%, tryptophan: 0.103%), HQPM1-B (lysine: 0.354%, tryptophan: 0.102%) and HQPM1-C (lysine: 0.347%, tryptophan: 0.105%) were superior in nutritional quality. HQPM4 had 0.334% lysine and 0.083% tryptophan, while the reconstituted hybrids viz., HQPM4-A (lysine: 0.388%, tryptophan: 0.097%), HQPM4-B (lysine: 0.400%, tryptophan: 0.098%) and HQPM4-C (lysine: 0.389%, tryptophan: 0.096%) possessed higher concentration of amino acids. The reconstituted hybrids of HQPM5 viz., HQPM5-A (lysine: 0.382%, tryptophan: 0.101%); HQPM5-B (lysine: 0.372%, tryptophan: 0.103%) and HQPM5-C (lysine: 0.383%, tryptophan: 0.104%) possessed higher nutritional value over the original hybrid, HQPM5 (lysine: 0.337%, tryptophan: 0.090%). Similarly, lysine and tryptophan concentration of HQPM7 was 0.361% and 0.093%, respectively while waxy version of the hybrids viz., HQPM7-A (lysine: 0.417%, tryptophan: 0.107%), HQPM7-B (lysine: 0.416%, tryptophan: 0.107%) and HQPM7-C (lysine: 0.412%, tryptophan: 0.107%) had higher accumulation (Figs. 6, 7). Across locations, reconstituted hybrids had 1.1-fold and 1.2-fold more lysine and tryptophan, respectively over the original hybrids.

### Evaluation of MAS-derived hybrids for yield and morphological characters

In general, reconstituted hybrids showed statistically similar grain yield, days to anthesis, days to silking, plant height and ear height with their original version (Table 6). The grain yield of reconstituted waxy QPM hybrids was 6248 kg/ha (range: 5724–7067 kg/ha), whereas in original QPM hybrids it was 6111 kg/ha (range: 5906–6648 kg/ha) across locations (Table 6). HQPM1 had grain yield of 5953 kg/ha, while its waxy hybrids had 6174 kg/ha (HQPM1-A), 6185 kg/ha (HQPM1-B), and 6056 kg/ha (HQPM1-C). Grain yield of the reconstituted hybrids was 5724 kg/ha (HQPM4-A), 5750 kg/ha (HQPM4-B), and 5971 kg/ha (HQPM4-C), compared to 5935 kg/ha in HQPM4. In case of HQPM5, grain yield was 5906 kg/ha, and its waxy reconstituted hybrids produced grain yield of 5968 kg/ha (HQPM5-A), 6102 kg/ha (HQPM5-B), and 5865 kg/ha (HQPM5-C). Similarly, HQPM7 had grain yield of 6648 kg/ha, whereas the waxy versions had grain yield of 7044 kg/ha (HQPM7-A), 7065 kg/ha (HQPM7-B), and 7067 kg/ha (HQPM7-C) (Table 6, Table S8). The flowering behaviour and plant characteristics of the reconstituted waxy hybrids were quite similar to the original hybrids as well (Table S8). However, in case of plant height and ear height, significant difference was observed between (1) HQPM1 and their reconstitute hybrids viz., HQPM1-C, and (2) HQPM4 and their reconstitute hybrids viz., HQPM1-B and HQPM1-C (Table 6). The improved waxy hybrids were very similar to their respective original hybrids for DUS characters except few traits (Table S9). For examples: anthocyanin colouration of brace root was present in HQPM1, while it was absent in HQPM1-A, HQPM1-B and HQPM1-C (Table S9a). Similarly, anthocyanin colouration of brace root was present in HQPM7, while it was found absent in HQPM7-A and HQPM7-C (Table S9d).

**Table 6:** Morphological characterization of reconstituted and original hybrids across locations.

S. no.	Hybrids	GY (kg/ha)	MF (days)	FF (days)	PH (cm)	EH (cm)
1	HQPM1	5953	49.8	52.5	174.8	86.3
2	HQPM1-A	6174	48.0	50.5	167.3	79.0
3	HQPM1-B	6185	47.7	50.5	175.1	81.2
4	HQPM1-C	6056	47.8	50.7	164.3	78.3
5	HQPM4	5935	50.2	52.8	173.6	86.8
6	HQPM4-A	5724	50.0	52.2	181.8	89.2
7	HQPM4-B	5750	49.3	51.8	190.3	95.5
8	HQPM4-C	5971	49.5	52.2	191.9	95.7
9	HQPM5	5906	49.7	52.5	176.3	90.2
10	HQPM5-A	5968	48.0	50.5	169.1	87.2
11	HQPM5-B	6102	49.0	51.7	171.5	80.2
12	HQPM5-C	5865	48.7	51.3	168.9	83.6
13	HQPM7	6648	50.3	53.2	170.7	85.8
14	HQPM7-A	7044	50.2	53.0	169.8	87.1
15	HQPM7-B	7065	49.2	51.7	178.5	84.2
16	HQPM7-C	7067	50.2	53.2	171.8	85.9
CD (5%)		518.68	2.81	2.64	8.79	7.06

*GY* Grain yield, *MF* days to anthesis, *FF* days to silking, *PH* plant height, *EH* ear height, A-, B-, C-: different versions, *CD *Critical difference

## Discussion

Waxy maize rich in amylopectin is highly popular in East and Southeast Asia^4,32^. Though large number of waxy maize cultivars are available for commercial cultivation worldwide^4^, waxy maize protein is poor in nutritional quality due to sub-optimal levels of essential amino acids like lysine and tryptophan^6,13^. Lack of waxy hybrids rich in lysine and tryptophan limits its great potential as a nutritious food to the resource poor especially in the developing countries^3^. Here, we used genomics-assisted breeding to combine high amylopectin, lysine and tryptophan in the genetic background of four popular sub-tropically adapted hybrids through marker-aided selection of recessive *wx1* and *o2* genes.

The gene-based markers viz., *wx-2507F/RG* and *phi057* helped in precisely selecting individual plants with favourable allele of both *wx1* and *o2* genes, respectively. Both the markers behaved co-dominantly and distinguished the homozygotes from heterozygotes^33^. Hossain et al.^3^ reported polymorphism among *Wx1* and *wx1* alleles using *wx-2507F/RG*. Zhang et al.^11^ observed polymorphism in *wx1* gene among recurrent and donor parents using gene-based SSRs viz., *phi027*, *phi061,* and *phi022*. While, Yang et al.^13^ reported *phi022* and *phi027* as polymorphic among the recurrent and donor parents. Several authors have also successfully used gene-based SSRs, *phi057* and *umc1066* to select *o2* allele in the MABB programme^22,26^. Identification of heterozygotes (BC_1_F_1_ and BC_2_F_1_) and homozygotes (BC_2_F_2_) at seedling stage helped in the exclusion of non-target progenies, resulting in significant savings of labour and material cost required for raising crops and pollination activities^29,34^. In the present study, *wx1* gene segregated as per Mendelian ratio of 1:1 in backcross generations and 1:2:1 in selfed generations. Yang et al.^13^ also reported 1:1 segregation in BC_1_F_1_ and BC_2_F_1_, while reported 1:2:1 ratio in F_2_ populations segregating for *wx1* gene. However, segregation distortion (SD) was observed for *o2* gene in some crosses. Similar observation was also observed by Jompuk et al.^35^ and Hossain et al.^22^ while analyzing the segregation of *o2* in various backcross populations. This SD could be caused by gametophytic factors, mutants such as faulty kernels, male sterility, and embryo-specific mutations^36^. SD warrants raising of large population size in order to achieve sufficient foreground positive genotypes in the MABB programme.

Since, *o2* and *wx1* genes are recessive, traditional backcross approach would have taken 12–14 seasons as each backcross generation would require progeny testing by selfing^22^. Two generation-based MABB, on the other hand, was efficient enough to generate comparable results in nearly half of the time (5–6 seasons). MABB strategy thus saved significant time and resources besides speeding up the breeding cycle^37^. Genomics-assisted background selection achieved high recovery of RPG in just two backcross generations^11,22^. The high recovery of RPG resulted in the phenotypic resemblance with their original versions. The introgressed inbreds and reconstituted hybrids possessed similar grain yield potential *at par* with the original versions. This was attributed to the selection recurrent parent alleles of SSRs linked to various loci relevant to yield attributing- and agronomic- characteristics^29^. The high recovery of RPG was further validated by great degree of similarity for the large number of DUS characters^38^. The difference for few traits between improved and original genotypes could be due to the fixation of donor allele or combination of genes from donor and recurrent parents^22^. However, few exceptions observed for the easily distinguishable morphological characteristics could be useful in registration and seed certification to differentiate newly derived genotypes from the original versions^39^.

Amylose is a linear homopolymer of glucopyranose units linked by α-(1,4) linkage, whereas amylopectin is a branched homopolymer of glucopyranose with both α-(1,4) and α-(1,6) linkages^9^. Introgressed inbreds and reconstituted hybrids recorded ~ 40% increase in amylopectin over original genotypes. Qi et al.^40^ also reported ~ 23% increase in amylopectin among waxy lines and hybrids (94.9%) compared to wild type genotypes (76.9%). Accumulation of higher amylopectin in waxy landraces and hybrids have also been reported by Stamp et al.^6^ Maize starch is composed of amylose and amylopectin fractions^3^. In maize, wild type *Wx1* codes functionally active GBSS-I that catalyzes the formation of amylose from ADP-glucose^41^. However, recessive *wx1* leads to impaired activity of GBSS-I which shifts the flux towards synthesis of amylopectin^32^. Mutant *wx1* results from various types of mutations including transposon/retroposon insertion and nucleotide deletion^42^. These mutations cause formation of premature stop codon or a change in amino acids in a critical region of the transcript, as well as splicing and translational mistakes^11^. Though MABB-derived *wx1*-based inbreds and reconstituted hybrids recorded enhanced amylopectin, they also exhibited moderate variation in amylopectin (95–99%) despite the presence of the identical *wx1* gene. This difference could be attributed to modifier loci or QTL that influence the accumulation of amylopectin in maize^9^. However, total starch content remained nearly same among the MABB-derived genotypes over their original versions. This suggested that increase in amylopectin among the *wx1*-based genotypes did not pose any negative effect on total starch content, which further justified the similar grain potential among the MAS-derived and their respective original versions.

MABB-derived lines and reconstituted hybrids having *o2* gene possessed higher lysine and tryptophan than the traditional maize^19^. Recessive *o2* leads to reduction of zein proteins (deficient in lysine and tryptophan), with a concurrent increase in non-zein proteins rich in lysine and tryptophan^43^. *o2* also down regulates the synthesis of lysine ketoglutarate reductase (LKR) resulting in increased levels of free lysine^44^. Besides, it is also involved in regulation of various lysine-rich proteins and enzymes^45^. However, *wx1wx1/o2o2*-based MABB derived inbreds and reconstituted hybrids possessed ~ 11–17% more lysine and tryptophan over the *o2o2*-based original genotypes. Zhou et al.^7^ introgressed *o2* gene into a waxy inbred (Zhao-OP-6/O2O2), and discovered that introgressed lines had 51.6% higher lysine than the original waxy line. Yang et al.^13^ also introgressed recessive *opaque16* (*o16*) gene from QCL3024 into two Chinese waxy lines, QCL5019 and QCL5008, and found that lysine content of the pyramid lines was 20% higher than the waxy parent. Zhang et al.^11^ further pyramided *o2* and *o16* in a waxy genetic background and found that pyramided lines (*wx1wx1/o2o2/o16o16*) accumulated 11% more lysine than *o2o2* genotypes. Thus, stacking of *wx1* and *o2* provided synergistic effects on accumulation lysine and tryptophan, which would provide better nutritional quality to alleviate malnutrition. Wang et al.^19^ analyzed RNA-sequencing of kernels (18th day after pollination) of *wx1wx1* and *o2o2/wx1wx1* inbreds, and revealed 49 differentially expressed genes (DEGs) related to mainly catalytic activity and metabolic processes. The *o2* gene regulated multiple metabolic pathways related to biological processes and molecular function during waxy maize endosperm development. In *o2o2/wx1wx1* line, the two genes that encode the EF-1α and LHT1 were up-regulated, and the gene that encodes sulfur-rich proteins was down-regulated, leading to the elevated levels of grain lysine^19^. Zhou et al.^7^ further compared *wx1wx1* inbred with *o2o2/wx1wx1* inbreds and concluded that *o2* introgression decreased the accumulation of various zein proteins and affected other endosperm proteins related to amino acid biosynthesis, starch-protein balance, stress response and signal transduction. Further, *wx1wx1/o2o2*-based inbreds and hybrids revealed moderate variation in lysine and tryptophan despite the presence of same *o2* allele. This variation is due to various modifier loci including *o16* that affect regulation of amino acid biosynthesis^29,46^.

Worldwide, white maize grains are preferred as human food over yellow maize^47^. White maize is also desirable in food-processing and corn-meal industries^48^. The predominance of white maize as food is due to various reasons that include cultural preference, organoleptic property and desire for the brightly coloured finished products^49–51^. People in East and South-East Asia also prefer white grained waxy maize^21,52^. Keeping this in view, it was important to develop white grained waxy maize hybrids, as yellow grained waxy maize is not preferred. Since, the recurrent parents were yellow in colour and donor line had white endosperm, it was possible to develop waxy inbreds and hybrids with white colour grains. The *Yellow1* (*Y1*) gene on chromosome-6 codes for *phytoene synthase* (*psy1*), which condenses two geranyl–geranyl pyrophosphate molecules into one molecule of phytoene in the carotenoid biosynthesis pathway^53^. The dominant *Y1* allele converts the step thereby leading to the synthesis of carotenoids and eventually yellow colour in the endosperm. However, the recessive *y1* allele is unable to catalyse the reaction and makes the kernel devoid of any carotenoids and eventually kernels look white^34^. In BC_2_F_3_ seeds borne on BC_2_F_2_ ears, *Y1* gene segregated in four forms viz., (i) dark yellow (*Y1Y1Y1*), medium yellow (*Y1Y1y1*), light yellow (*Y1y1y1*) and white (*y1y1y1*) in the endosperm^54^. We selected only the white kernels to raise the BC_2_F_3_ progenies, and eventually develop white grained waxy hybrids.

These newly derived white waxy hybrids possess diverse usage as food and various industrial products^3^. Globally immature waxy maize ears are gaining popularity as a breakfast item. It is also widely used to improve the viscosity, freeze–thaw stability, uniformity, and appearance of the food products^55^. Due to high amylopectin content, food made from waxy maize is easily digested in the human gut^10,56^. Amylopectin powder is a preferred food after workout in gym and body building industry^8^. Further, pure amylopectin powder possesses special pasting properties, thus used as a popular ingredient in textile, adhesive and paper industries^32^. Since waxy maize starch has a higher hydrolysis rate, it has higher starch-to-ethanol conversion efficiency when used to make ethanol^57^.

Further, these white waxy hybrids are also rich in lysine and tryptophan, thus possess superior protein quality. So far, large number of QPM hybrids rich in lysine and tryptophan have been developed and commercialized worldwide^58^. But these QPM hybrids do not possess high amount of amylopectin^11^. On the other hand, several waxy landrace and hybrids have been in cultivation especially in East- and Sout-East Asian countries^4^. These waxy cultivars are poor in nutritional quality as they lack required amount of lysine and tryptophan^13^. Though few studies have improved *wx1* inbreds for nutritional qualities, the present study possesses novelty on three aspects, viz*.* (1) studies by Yang et al. (2013), Zhou et al. (2016) and Zhang et al. (2013) have mentioned the enhancement of only lysine, but we analyzed the effects on both lysine and tryptophan among the waxy genotypes. These two are the essential amino acids not synthesized in our body, thus possess paramount importance for growth and development in humans, (2) earlier studies have analyzed the levels of amylopectin and lysine only in inbreds, but here we combined *wx1* and *o2* genes in elite inbreds, and further developed and evaluated the performance of hybrids for amylopectin, lysine, tryptophan, grain yield, and agronomic performance, and (3) previous studies have combined *wx1* and *o2* genes in temperate background, while lines in the present study are sub-tropically adapted. These newly derived waxy hybrids with superior protein quality would help in providing the balanced diet and alleviate the malnutrition in a sustainable and cost-efficient manner^59^. These nutritious waxy hybrids are also high yielding and would help the farmers to earn livelihood. The present investigation is the first report development of waxy hybrids rich in lysine and tryptophan using accelerated-breeding strategy.

## Conclusions

Waxy maize rich in amylopectin is becoming increasingly important as a source of human nutrition, livelihood, and income generation. However, their usage as a preferred food and industrial product is limited due to lack of suitable waxy hybrids. Here, we have developed four high yielding waxy hybrids rich in amylopectin. These waxy hybrids also possess quality protein, besides high grain yield. The improved waxy QPM hybrids developed in this study can be directly commercialized and used for human consumption. Further, the improved waxy QPM maize inbreds will serve as potential donors for the development of the lysine and tryptophan rich waxy hybrids in the breeding programmes. This is the first report of development of maize hybrids rich in amylopectin, lysine and tryptophan.

## Supplementary Information

Supplementary Information.
