# Supplementary material for: Combining higher accumulation of amylopectin, lysine and tryptophan in maize hybrids through genomics-assisted stacking of waxy1 and opaque2 genes
Source: Sci Rep. 2022 Jan 13;12:706. doi: 10.1038/s41598-021-04698-3 (PMC8758761; doi:10.1038/s41598-021-04698-3)
Supplement: Supplementary file 1 — Supplementary Information. [file 41598_2021_4698_MOESM1_ESM.pdf]

Combining higher accumulation of amylopectin, lysine and tryptophan in maize hybrids through genomics-assisted stacking of *waxy1* and *opaque2* genes

Zahirul A. Talukder[1], Vignesh Muthusamy[1], Rashmi Chhabra[1], Nisrita Gain[1], Shashidhar B. Reddappa[1], Subhra J. Mishra[1], Ravindra Kasana[1], Vinay Bhatt[1], Gulab Chand[1], Ashvinkumar Katral[1], Brijesh Mehta[2], Satish K. Guleria[3], Rajkumar U. Zunjare[1] and Firoz Hossain[1]*

[1]ICAR-Indian Agricultural Research Institute (IARI), New Delhi, India; [2]ICAR-Indian Grassland and Fodder Research Institute (IGFRI), Jhansi, India; [3]CSK-Himachal Pradesh Krishi Vishvavidyalaya (CSK-HPKV), Bajaura, India

**Corresponding author: fh_gpb@yahoo.com*

Table S1: Details of popular commercial hybrids targeted for amylopectin enrichment

S. No.	Hybrids	Parental combination	Maturity group	Maturity (days)	Area of adaption
1	HQPM1	HKI193-1 × HKII63	Medium	88-90	Across the country
2	HQPM4	HKI193-2 × HKII61	Medium	95-97	Across the country
3	HQPM5	HKI163 × HKII61	Medium	92-93	Across the country
4	HQPM7	HKI193-1 × HKII61	Medium	96-97	Peninsular zone

Table S2. Details of genetic materials used in the present study

S. No.	Genotypes	Derived from	Kernel colour	Maturity	Institution
Recurrent parents					
1.	HKI161	CML161 (P25QPM)	Yellow	Medium	CCSHAU, Uchani
2.	HKI163	CML163 (P26QPM)	Yellow	Medium	CCSHAU, Uchani
3.	HKI193-1	CML193	Yellow	Medium	CCSHAU, Uchani
4	HKI193-2	CML193	Yellow	Medium	CCSHAU, Uchani
Donor parent					
5.	MGU-102-*wx1*	CIMMYT population	White	Medium	IARI, New Delhi

Table S3. Details of parents, hybrids and backcross- and self- progenies generated under MABB

S. No.	Generation	Season	Location
1.	P_1 and P_2	rainy (2016)	IARI, Delhi
2.	F_1	winter (2016-17)	IIMR-WNC, Hyderabad
3.	BC_1F_1	rainy (2017)	IARI, Delhi
4.	BC_2F_1	winter (2017-18)	IIMR-WNC, Hyderabad
5.	BC_2F_2	rainy (2018)	IARI, Delhi
6.	BC_2F_3	rainy (2019)	IARI, Delhi
7.	Generation of F_1 hybrids	winter (2019-20)	IIMR-WNC, Hyderabad
8.	Evaluation of reconstituted hybrids	rainy (2020)	IARI, Delhi, CSK-HPKV, Bajaura & IGFRI, Jhansi

Table S4. Details of gene-based marker used for foreground selection

S. No.	Gene	Marker	Type	Primer sequence ($5^{/}$-$3^{/}$)	Reference
1.	*waxy1*	*wx-2507F/RG*	InDel	F: ACCTCAAGAGCAACTACCAGTC<br>R: AAGGACGACTTGAATCTCTCC	Shin et al. 2006
2.	*opaque2*	*phi057*	SSR	F: CTCATCAGTGCCGTCGTCCAT<br>R: CAGTCGCAAGAAACCGTTGCC	Gupta et al. 2013

F; forward, R: reverse

Table S5: Starch content among MAS derived inbreds and hybrids along with their original versions

S. No.	Genotypes	Starch (%)	Hybrids	Starch (%)
1.	HKI161	67.38	HQPM1	69.98
2.	HKI161-99-30-43-290	69.10	HQPM1-A-W	71.87
3.	HKI161-107-42-1-291	68.80	HQPM1-B-W	70.95
4.	HKI161-107-42-10-293	68.90	HQPM1-C-W	71.53
5.	HKI163	69.46	HQPM4	68.92
6.	HKI163-9-2-13-302	69.90	HQPM4-A-W	71.03
7.	HKI163-9-35-88-303	70.30	HQPM4-B-W	71.21
8.	HKI163-19-3-107-304	71.26	HQPM4-C-W	70.74
9.	HKI193-1	68.19	HQPM5	71.13
10.	HKI193-1-6-55-9-317	70.40	HQPM5-A-W	72.59
11.	HKI193-1-6-55-116-319	68.90	HQPM5-B-W	72.09
12.	HKI193-1-14-1-57-320	69.40	HQPM5-C-W	72.80
13.	HKI193-2	67.18	HQPM7	70.78
14.	HKI193-2-4-39-45-321	69.23	HQPM7-A-W	71.53
15.	HKI193-2-4-20-56-322	67.94	HQPM7-B-W	71.35
16.	HKI193-2-4-20-111-325	68.85	HQPM7-C-W	72.18
17.	CD (5%)	11.05		12.64

Table S6. Amylopectin, lysine and tryptophan content in original- and reconstituted waxy- hybrids

S. No.	Genotypes	Amylopectin (%)	Lysine (%)	Tryptophan (%)
1.	HQPM1	71.6	0.314	0.091
2.	HQPM1-A	98.1	0.353	0.103
3.	HQPM1-B	98.5	0.354	0.102
4.	HQPM1-C	98.8	0.347	0.105
5.	HQPM4	70.4	0.334	0.083
6.	HQPM4-A	99.0	0.388	0.097
7.	HQPM4-B	98.4	0.400	0.098
8.	HQPM4-C	98.8	0.389	0.096
9.	HQPM5	73.4	0.337	0.090
10.	HQPM5-A	99.1	0.382	0.101
11.	HQPM5-B	98.9	0.372	0.103
12.	HQPM5-C	99.0	0.383	0.104
13.	HQPM7	74.4	0.361	0.093
14.	HQPM7-A	99.0	0.417	0.107
15.	HQPM7-B	99.4	0.416	0.107
16.	HQPM7-C	99.1	0.412	0.107
	CD (5%)	5.45	0.02	0.004

Table S7a. DUS characteristics of HKI161 and its MABB-derived progenies

S. No.	Characteristics	HKI161	HKI161-99-30-43-290	HKI161-107-42-1-291	HKI161-107-42-10-293
		Yellow kernel	White kernel	White kernel	White kernel
1.	Leaf: angle between blade and stem (on leaf just above upper ear)	Wide	Wide	Wide	Wide
2.	Leaf: attitude of blade	Drooping	Drooping	Drooping	Drooping
3.	Stem: anthocyanin colouration of brace root	Present	Absent	Absent	Absent
4.	Tassel: time of anthesis	Medium	Medium	Medium	Medium
5.	Tassel: anthocyanin colouration at base of glume	Absent	Absent	Absent	Absent
6.	Tassel: anthocyanin colouration of glumes excluding base	Present	Absent	Present	Absent
7.	Tassel: anthocyanin colouration of anthers	Present	Present	Present	Present
8.	Tassel: density of spikelets	Dense	Dense	Dense	Dense
9.	Tassel: angle between main axis and lateral branches	Narrow	Narrow	Narrow	Narrow
10.	Tassel: attitude of lateral branches	Straight	Straight	Straight	Straight
11.	Ear: time of silk emergence	Medium	Medium	Medium	Medium
12.	Ear: anthocyanin colouration of silks	Present	Present	Present	Present
13.	Leaf: anthocyanin colouration of sheath	Present	Present	Absent	Absent
14.	Tassel: length of main axis above lowest side branch	Medium	Medium	Medium	Medium
15.	Plant length up to flag leaf	Long	Long	Long	Long
16.	Plant: ear placement	Medium	Medium	Medium	Medium
17.	Leaf: width of blade	Medium	Medium	Medium	Medium
18.	Ear: length without husk	Medium	Medium	Medium	Medium
19.	Ear: diameter	Small	Small	Small	Small
20.	Ear: shape	Conico-cylindrical	Conico-cylinderical	Conico-cylinderical	Conico-cylinderical
21.	Ear: number of rows of grains	Medium	Medium	Medium	Medium
22.	Ear: type of grain	Flint	Flint	Flint	Flint
23.	Ear: colour of top of grain	Orange	White	White	White
24.	Ear: colouration of glumes of cob	Absent	Absent	Absent	Absent
25.	Kernel: row arrangement	Straight	Straight	Straight	Straight
26.	Kernel: poppiness	Absent	Absent	Absent	Absent

27.	Kernel: sweetness	Absent	Absent	Absent	Absent
28.	Kernel: waxiness	Absent	Present	Present	Present
29.	Kernel: opaqueness	Present	Present	Present	Present
30.	Kernel: shape	Round	Round	Round	Round
31.	Kernel: 1000 kernel weight	Large	Large	Large	Large

Table S7b. DUS characteristics of HKI163 and its MABB-derived progenies

S. No.	Characteristics	HKI163	HKI163-9-2-13-302	HKI161-107-42-1-291	HKI161-107-42-10-293
		Yellow kernel	White kernel	White kernel	White kernel
1.	Leaf: angle between blade and stem (on leaf just above upper ear)	Small	Wide	Wide	Wide
2.	Leaf: attitude of blade	Straight	Drooping	Drooping	Drooping
3.	Stem: anthocyanin colouration of brace root	Present	Present	Present	Present
4.	Tassel: time of anthesis	Late	Late	Late	Late
5.	Tassel: anthocyanin colouration at base of glume	Absent	Absent	Absent	Absent
6.	Tassel: anthocyanin colouration of glumes excluding base	Absent	Absent	Absent	Absent
7.	Tassel: anthocyanin colouration of anthers	Absent	Absent	Absent	Absent
8.	Tassel: density of spikelets	Dense	Dense	Dense	Dense
9.	Tassel: angle between main axis and lateral branches	Narrow	Narrow	Narrow	Narrow
10.	Tassel: attitude of lateral branches	Straight	Straight	Straight	Straight
11.	Ear: time of silk emergence	Late	Medium	Medium	Medium
12.	Ear: anthocyanin colouration of silks	Present	Present	Absent	Absent
13.	Leaf: anthocyanin colouration of sheath	Absent	Absent	Absent	Absent
14.	Tassel: length of main axis above lowest side branch	Medium	Medium	Medium	Medium
15.	Plant length up to flag leaf	Long	Long	Long	Long
16.	Plant: ear placement	Medium	Medium	Medium	Medium
17.	Leaf: width of blade	Medium	Medium	Medium	Medium
18.	Ear: length without husk	Medium	Medium	Medium	Medium
19.	Ear: diameter	Small	Small	Small	Small
20.	Ear: shape	Conico-cylindrical	Conico-cylinderical	Conico-cylinderical	Conico-cylinderical
21.	Ear: number of rows of grains	Medium	Medium	Medium	Medium
22.	Ear: type of grain	Semi-flint	Semi-flint	Semi-flint	Semi-flint
23.	Ear: colour of top of grain	Yellow	White	White	White
24.	Ear: colouration of glumes of cob	Absent	Absent	Absent	Absent
25.	Kernel: row arrangement	Straight	Straight	Straight	Straight
26.	Kernel: poppiness	Absent	Absent	Absent	Absent
27.	Kernel: sweetness	Absent	Absent	Absent	Absent
28.	Kernel: waxiness	Absent	Present	Present	Present
29.	Kernel: opaqueness	Present	Present	Present	Present

30.	Kernel: shape	Round	Round	Round	Round
31.	Kernel: 1000 kernel weight	Medium	Medium	Medium	Medium

Table S7c. DUS characteristics of HKI193-1 and its MABB-derived progenies

S. No.	Characteristics	HKI193-1	HKI193-1-6-55-9-317	HKI193-1-6-55-116-319	HKI193-1-14-1-57-320
		Yellow kernel	White kernel	White kernel	White kernel
1.	Leaf: angle between blade and stem (on leaf just above upper ear)	Wide	Wide	Wide	Wide
2.	Leaf: attitude of blade	Drooping	Drooping	Drooping	Drooping
3.	Stem: anthocyanin colouration of brace root	Absent	Present	Present	Present
4.	Tassel: time of anthesis	Late	Medium	Medium	Medium
5.	Tassel: anthocyanin colouration at base of glume	Absent	Absent	Absent	Absent
6.	Tassel: anthocyanin colouration of glumes excluding base	Absent	Absent	Absent	Absent
7.	Tassel: anthocyanin colouration of anthers	Present	Present	Present	Present
8.	Tassel: density of spikelets	Dense	Dense	Dense	Dense
9.	Tassel: angle between main axis and lateral branches	Narrow	Narrow	Narrow	Narrow
10.	Tassel: attitude of lateral branches	Straight	Straight	Straight	Straight
11.	Ear: time of silk emergence	Late	Medium	Medium	Medium
12.	Ear: anthocyanin colouration of silks	Absent	Absent	Absent	Absent
13.	Leaf: anthocyanin colouration of sheath	Present	Absent	Absent	Absent
14.	Tassel: length of main axis above lowest side branch	Medium	Medium	Medium	Medium
15.	Plant length up to flag leaf	Long	Long	Long	Long
16.	Plant: ear placement	Medium	Medium	Medium	Medium
17.	Leaf: width of blade	Medium	Medium	Medium	Medium
18.	Ear: length without husk	Medium	Medium	Medium	Medium
19.	Ear: diameter	Small	Small	Small	Small
20.	Ear: shape	Conico-Cylindrical	Conico-Cylinderical	Conico-Cylinderical	Conico-Cylinderical
21.	Ear: number of rows of grains	Medium	Medium	Medium	Medium
22.	Ear: type of grain	Semi-flint	Semi-flint	Semi-flint	Semi-flint
23.	Ear: colour of top of grain	Yellow	White	White	White

24.	Ear: colouration of glumes of cob	Absent	Absent	Absent	Absent
25.	Kernel: row arrangement	Straight	Straight	Straight	Straight
26.	Kernel: poppiness	Absent	Absent	Absent	Absent
27.	Kernel: sweetness	Absent	Absent	Absent	Absent
28.	Kernel: waxiness	Absent	Present	Present	Present
29.	Kernel: opaqueness	Present	Present	Present	Present
30.	Kernel: shape	Round	Round	Round	Round
31.	Kernel: 1000 kernel weight	Small	Medium	Medium	Medium

Table S7d. DUS characteristics of HKI93-2 and its MABB-derived progenies

S. No.	Characteristics	HKI93-2	HKI193-2-4-39-45-321	HKI193-2-4-20-56-322	HKI193-2-4-20-111-325
		Yellow kernel	White kernel	White kernel	White kernel
1.	Leaf: angle between blade and stem (on leaf just above upper ear)	Small	Small	Small	Small
2.	Leaf: attitude of blade	Drooping	Drooping	Drooping	Drooping
3.	Stem: anthocyanin colouration of brace root	Absent	Absent	Absent	Absent
4.	Tassel: time of anthesis	Medium	Medium	Medium	Medium
5.	Tassel: anthocyanin colouration at base of glume	Present	Absent	Absent	Absent
6.	Tassel: anthocyanin colouration of glumes excluding base	Absent	Absent	Absent	Absent
7.	Tassel: anthocyanin colouration of anthers	Present	Present	Present	Present
8.	Tassel: density of spikelets	Dense	Dense	Dense	Dense
9.	Tassel: angle between main axis and lateral branches	Narrow	Narrow	Narrow	Narrow
10.	Tassel: attitude of lateral branches	Straight	Straight	Straight	Straight
11.	Ear: time of silk emergence	Medium	Medium	Medium	Medium
12.	Ear: anthocyanin colouration of silks	Absent	Absent	Absent	Absent
13.	Leaf: anthocyanin colouration of sheath	Absent	Absent	Absent	Absent
14.	Tassel: length of main axis above lowest side branch	Medium	Medium	Medium	Medium
15.	Plant length up to flag leaf	Long	Long	Long	Long
16.	Plant: ear placement	Medium	Medium	Medium	Medium
17.	Leaf: width of blade	Medium	Medium	Medium	Medium
18.	Ear: length without husk	Medium	Medium	Medium	Medium
19.	Ear: diameter	Small	Small	Small	Small

20.	Ear: shape	Conico-cylinderical	Conico-cylinderical	Conico-cylinderical	Conico-cylinderical
21.	Ear: number of rows of grains	Medium	Medium	Medium	Medium
22.	Ear: type of grain	Semi dent	Semi dent	Semi dent	Semi dent
23.	Ear: colour of top of grain	White	White	White	White
24.	Ear: colouration of glumes of cob	Absent	Absent	Absent	Absent
25.	Kernel: row arrangement	Straight	Straight	Straight	Straight
26.	Kernel: poppiness	Absent	Absent	Absent	Absent
27.	Kernel: sweetness	Absent	Absent	Absent	Absent
28.	Kernel: waxiness	Absent	Present	Present	Present
29.	Kernel: opaqueness	Present	Present	Present	Present
30.	Kernel: shape	Toothed	Toothed	Toothed	Toothed
31.	Kernel: 1000 kernel weight	Medium	Medium	Medium	Medium

Table S8a. Morphological characterization of reconstituted hybrids along with their original versions

S. No.	Hybrids	Grain yield (kg/ha)			Days to 50% male flowering (days)			Days to 50% female flowering (days)		
		Delhi	Bajaura	Jhansi	Delhi	Bajaura	Jhansi	Delhi	Bajaura	Jhansi
1.	HQPM1	5755	6297	5806	45.5	58.5	45.5	48.0	61.0	48.5
2.	HQPM1-A-W	5917	6542	6062	44.5	53.0	46.5	47.0	55.0	49.5
3.	HQPM1-B-W	6047	6736	5771	44.5	52.0	46.5	47.5	54.5	49.5
4.	HQPM1-C-W	6118	6458	5591	44.5	53.0	46.0	47.5	55.5	49.0
5.	HQPM4	5911	6144	5749	45.0	58.0	47.5	47.5	60.5	50.5
6.	HQPM4-A-W	5630	6038	5502	46.0	55.5	48.5	48.0	58.0	50.5
7.	HQPM4-B-W	5447	6354	5449	45.0	55.0	48.0	47.5	57.0	51.0
8.	HQPM4-C-W	5730	6177	6005	45.0	56.5	47.0	48.0	58.5	50.0
9.	HQPM5	5708	6433	5577	45.5	56.0	47.5	48.5	58.5	50.5
10.	HQPM5-A-W	5964	6244	5698	45.0	52.5	46.5	47.0	55.0	49.5
11.	HQPM5-B-W	5817	6522	5968	45.0	53.0	49.0	48.0	55.0	52.0
12.	HQPM5-C-W	5499	6726	5371	45.0	53.0	48.0	47.5	56.0	50.5
13.	HQPM7	6820	6871	6252	46.0	57.5	47.5	48.5	60.5	50.5
14.	HQPM7-A-W	7190	7467	6475	48.0	54.0	48.5	51.0	56.5	51.5
15.	HQPM7-B-W	7231	7539	6425	45.0	54.5	48.0	48.0	56.5	50.5
16.	HQPM7-C-W	7131	7704	6364	46.0	54.5	50.0	49.0	58.0	52.5
17.	CD (5%)	635.3	460.07	580.02	3.05	3.69	1.82	2.91	3.39	1.98

CD: Critical difference

Table S8b. Morphological characterization of reconstituted hybrids along with their original versions

S. No.	Hybrids	Plant height (cm)			Ear height (cm)		
		Delhi	Bajaura	Jhansi	Delhi	Bajaura	Jhansi
1.	HQPM1	174.5	181.2	168.8	85.3	93.0	85.3
2.	HQPM1-A-W	162.0	173.5	166.5	82.8	81.8	82.8
3.	HQPM1-B-W	169.7	182.3	173.3	89.0	85.1	89.0
4.	HQPM1-C-W	158.2	170.0	164.7	83.7	76.4	83.7
5.	HQPM4	173.0	174.5	173.3	87.7	92.7	87.7
6.	HQPM4-A-W	181.7	188.3	175.5	86.0	92.7	86.0
7.	HQPM4-B-W	188.0	200.8	182.2	94.7	95.9	94.7
8.	HQPM4-C-W	195.8	197.2	182.7	94.2	94.3	94.2
9.	HQPM5	167.8	186.3	174.8	90.8	91.6	90.8
10.	HQPM5-A-W	169.3	157.7	180.2	95.2	79.0	95.2
11.	HQPM5-B-W	171.5	173.5	169.5	86.5	75.8	86.5
12.	HQPM5-C-W	160.3	176.3	170.2	78.0	91.8	78.0
13.	HQPM7	168.8	175.8	167.5	82.5	90.0	82.5
14.	HQPM7-A-W	160.7	180.0	168.7	86.8	86.2	86.8
15.	HQPM7-B-W	178.5	184.0	173.0	85.0	82.7	85.0
16.	HQPM7-C-W	166.3	179.5	169.7	82.5	92.6	82.5
17.	CD (5%)	11.45	10.13	9.65	12.24	9.49	9.80

CD: Critical difference

Table S9a. DUS characteristics of HQPM1 and its reconstituted versions

S. No.	Characteristics	HQPM1	HQPM1-A	HQPM1-B	HQPM1-C
		Yellow kernel	White kernel	White kernel	White kernel
1.	Leaf: angle between blade and stem (on leaf just above upper ear)	Wide	Wide	Wide	Wide
2.	Leaf: attitude of blade	Drooping	Drooping	Drooping	Drooping
3.	Stem: anthocyanin colouration of brace root	Present	Absent	Absent	Absent
4.	Tassel: time of anthesis	Late	Medium	Medium	Medium
5.	Tassel: anthocyanin colouration at base of glume	Absent	Absent	Absent	Absent
6.	Tassel: anthocyanin colouration of glumes excluding base	Absent	Absent	Absent	Absent
7.	Tassel: anthocyanin colouration of anthers	Present	Present	Present	Present
8.	Tassel: density of spikelets	Dense	Dense	Dense	Dense
9.	Tassel: angle between main axis and lateral branches	Narrow	Narrow	Narrow	Narrow
10.	Tassel: attitude of lateral branches	Straight	Straight	Straight	Straight
11.	Ear: time of silk emergence	Late	Medium	Medium	Medium
12.	Ear: anthocyanin colouration of silks	Absent	Absent	Absent	Absent
13.	Leaf: anthocyanin colouration of sheath	Absent	Absent	Absent	Absent
14.	Tassel: length of main axis above lowest side branch	Medium	Medium	Medium	Medium
15.	Plant length up to flag leaf	Long	Long	Long	Long
16.	Plant: ear placement	Medium	Medium	Medium	Medium
17.	Leaf: width of blade	Medium	Medium	Medium	Medium
18.	Ear: length without husk	Long	Long	Long	Long
19.	Ear: diameter	Medium	Medium	Medium	Medium
20.	Ear: shape	Conico-cylindrical	Conico-cylinderical	Conico-cylinderical	Conico-cylinderical
21.	Ear: number of rows of grains	Many	Many	Many	Many
22.	Ear: type of grain	Semi-dent	Semi-dent	Semi-dent	Semi-dent
23.	Ear: colour of top of grain	Yellow	White	White	White
24.	Ear: colouration of glumes of cob	Absent	Absent	Absent	Absent
25.	Kernel: row arrangement	Straight	Straight	Straight	Straight
26.	Kernel: poppiness	Absent	Absent	Absent	Absent
27.	Kernel: sweetness	Absent	Absent	Absent	Absent
28.	Kernel: waxiness	Absent	Present	Present	Present

29.	Kernel: opaqueness	Present	Present	Present	Present
30.	Kernel: shape	Round	Round	Round	Round
31.	Kernel: 1000 kernel weight	Large	Medium	Medium	Medium

Table S9b. DUS characteristics of HQPM4 and its reconstituted versions

S. No.	Characteristics	HQPM4	HQPM4-A	HQPM4-B	HQPM4-C
		Yellow kernel	White kernel	White kernel	White kernel
1.	Leaf: angle between blade and stem (on leaf just above upper ear)	Wide	Wide	Wide	Wide
2.	Leaf: attitude of blade	Drooping	Drooping	Drooping	Drooping
3.	Stem: anthocyanin colouration of brace root	Present	Absent	Absent	Present
4.	Tassel: time of anthesis	Medium	Early	Early	Early
5.	Tassel: anthocyanin colouration at base of glume	Absent	Absent	Absent	Absent
6.	Tassel: anthocyanin colouration of glumes excluding base	Absent	Absent	Absent	Present
7.	Tassel: anthocyanin colouration of anthers	Present	Present	Present	Present
8.	Tassel: density of spikelets	Dense	Dense	Dense	Dense
9.	Tassel: angle between main axis and lateral branches	Wide	Wide	Wide	Wide
10.	Tassel: attitude of lateral branches	Curved	Curved	Curved	Curved
11.	Ear: time of silk emergence	Medium	Early	Early	Early
12.	Ear: anthocyanin colouration of silks	Present	Absent	Absent	Present
13.	Leaf: anthocyanin colouration of sheath	Absent	Absent	Absent	Absent
14.	Tassel: length of main axis above lowest side branch	Medium	Medium	Medium	Medium
15.	Plant length up to flag leaf	Long	Long	Long	Long
16.	Plant: ear placement	Medium	Medium	Medium	Medium
17.	Leaf: width of blade	Broad	Broad	Broad	Broad
18.	Ear: length without husk	Long	Long	Long	Long
19.	Ear: diameter	Large	Large	Large	Large
20.	Ear: shape	Conico-cylinderical	Conico-cylinderical	Conico-cylinderical	Conico-cylinderical
21.	Ear: number of rows of grains	Many	Many	Many	Many
22.	Ear: type of grain	Flint	Flint	Flint	Flint
23.	Ear: colour of top of grain	Yellow wih cap	White	White	White
24.	Ear: colouration of glumes of cob	Absent	White	White	White

25.	Kernel: row arrangement	Straight	Straight	Straight	Straight
26.	Kernel: poppiness	Absent	Absent	Absent	Absent
27.	Kernel: sweetness	Absent	Absent	Absent	Absent
28.	Kernel: waxiness	Absent	Present	Present	Present
29.	Kernel: opaqueness	Present	Present	Present	Present
30.	Kernel: shape	Round	Round	Round	Round
31.	Kernel: 1000 kernel weight	Medium	Medium	Medium	Medium

Table S9c. DUS characteristics of HQPM5 and its reconstitut versions

S. No.	Characteristics	HQPM5	HQPM5-A	HQPM5-B	HQPM5-C
		Yellow kernel	White kernel		
1.	Leaf: angle between blade and stem (on leaf just above upper ear)	Wide	Wide	Wide	Wide
2.	Leaf: attitude of blade	Drooping	Drooping	Drooping	Drooping
3.	Stem: anthocyanin colouration of brace root	Present	Absent	Present	Present
4.	Tassel: time of anthesis	Medium	Early	Early	Early
5.	Tassel: anthocyanin colouration at base of glume	Absent	Absent	Absent	Absent
6.	Tassel: anthocyanin colouration of glumes excluding base	Present	Present	Present	Present
7.	Tassel: anthocyanin colouration of anthers	Present	Present	Present	Present
8.	Tassel: density of spikelets	Dense	Dense	Dense	Dense
9.	Tassel: angle between main axis and lateral branches	Narrow	Narrow	Narrow	Narrow
10.	Tassel: attitude of lateral branches	Straight	Curved	Curved	Curved
11.	Ear: time of silk emergence	Medium	Straight	Straight	Straight
12.	Ear: anthocyanin colouration of silks	Present	Present	Present	Present
13.	Leaf: anthocyanin colouration of sheath	Present	Absent	Present	Absent
14.	Tassel: length of main axis above lowest side branch	Long	Long	Long	Long
15.	Plant length up to flag leaf	Long	Long	Long	Long
16.	Plant: ear placement	Medium	Medium	Medium	Medium
17.	Leaf: width of blade	Medium	Medium	Medium	Medium
18.	Ear: length without husk	Long	Long	Long	Long
19.	Ear: diameter	Medium	Medium	Medium	Medium
20.	Ear: shape	Conico-cylindrical	Conico-cylinderical	Conico-cylinderical	Conico-cylinderical

21.	Ear: number of rows of grains	Many	Many	Many	Many
22.	Ear: type of grain	Semi-flint	Semi-flint	Semi-flint	Semi-flint
23.	Ear: colour of top of grain	Orange	White wih cap	White wih cap	White wih cap
24.	Ear: colouration of glumes of cob	Absent	Absent	Absent	Absent
25.	Kernel: row arrangement	Straight	Straight	Straight	Straight
26.	Kernel: poppiness	Absent	Absent	Absent	Absent
27.	Kernel: sweetness	Absent	Absent	Absent	Absent
28.	Kernel: waxiness	Absent	Present	Present	Present
29.	Kernel: opaqueness	Present	Present	Present	Present
30.	Kernel: shape	Round	Round	Round	Round
31.	Kernel: 1000 kernel weight	Large	Large	Large	Large

Table S9d. DUS characteristics of HQPM7 and its reconstituted versions

S. No.	Characteristics	HQPM7	HQPM7-A	HQPM7-B	HQPM7-C
		Yellow kernel	White kernel	White kernel	White kernel
1.	Leaf: angle between blade and stem (on leaf just above upper ear)	Wide	Wide	Wide	Wide
2.	Leaf: attitude of blade	Drooping	Drooping	Drooping	Drooping
3.	Stem: anthocyanin colouration of brace root	Present	Absent	Present	Absent
4.	Tassel: time of anthesis	Medium	Early	Early	Early
5.	Tassel: anthocyanin colouration at base of glume	Absent	Absent	Absent	Absent
6.	Tassel: anthocyanin colouration of glumes excluding base	Present	Absent	Absent	Absent
7.	Tassel: anthocyanin colouration of anthers	Present	Absent	Present	Absent
8.	Tassel: density of spikelets	Dense	Dense	Dense	Dense
9.	Tassel: angle between main axis and lateral branches	Narrow	Narrow	Narrow	Narrow
10.	Tassel: attitude of lateral branches	Straight	Straight	Straight	Straight
11.	Ear: time of silk emergence	Medium	Early	Early	Early
12.	Ear: anthocyanin colouration of silks	Present	Absent	Present	Absent
13.	Leaf: anthocyanin colouration of sheath	Present	Absent	Absent	Absent
14.	Tassel: length of main axis above lowest side branch	Long	Long	Long	Long
15.	Plant length up to flag leaf	Long	Long	Long	Long
16.	Plant: ear placement	Medium	Medium	Medium	Medium
17.	Leaf: width of blade	Medium	Medium	Medium	Medium

18.	Ear: length without husk	Long	Long	Long	Long
19.	Ear: diameter	Medium	Medium	Medium	Medium
20.	Ear: shape	Conico-cylindrical	Conico-cylindrical	Conico-cylindrical	Conico-cylindrical
21.	Ear: number of rows of grains	Many	Many	Many	Many
22.	Ear: type of grain	Semi-flint	Semi-flint	Semi-flint	Semi-flint
23.	Ear: colour of top of grain	Orange	White with cap	White with cap	White with cap
24.	Ear: colouration of glumes of cob	Absent	Absent	Absent	Absent
25.	Kernel: row arrangement	Straight	Straight	Straight	Straight
26.	Kernel: poppiness	Absent	Absent	Absent	Absent
27.	Kernel: sweetness	Absent	Absent	Absent	Absent
28.	Kernel: waxiness	Absent	Present	Present	Present
29.	Kernel: opaqueness	Present	Present	Present	Present
30.	Kernel: shape	Round	Round	Round	Round
31.	Kernel: 1000 kernel weight	Large	Large	Large	Large

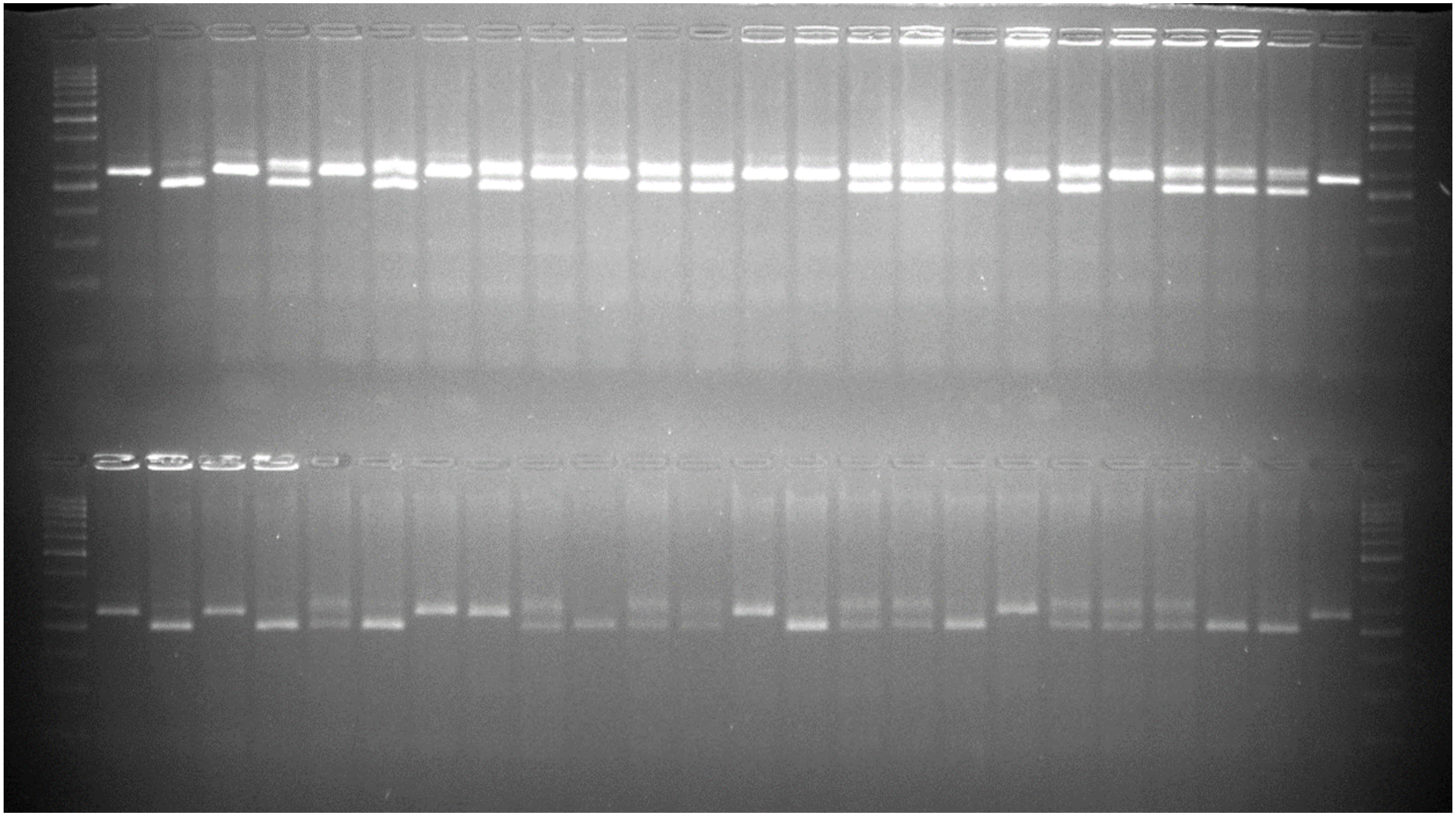

Fig 1S: Original gel image of foreground selection of wx1 gene as given in Fig 1.

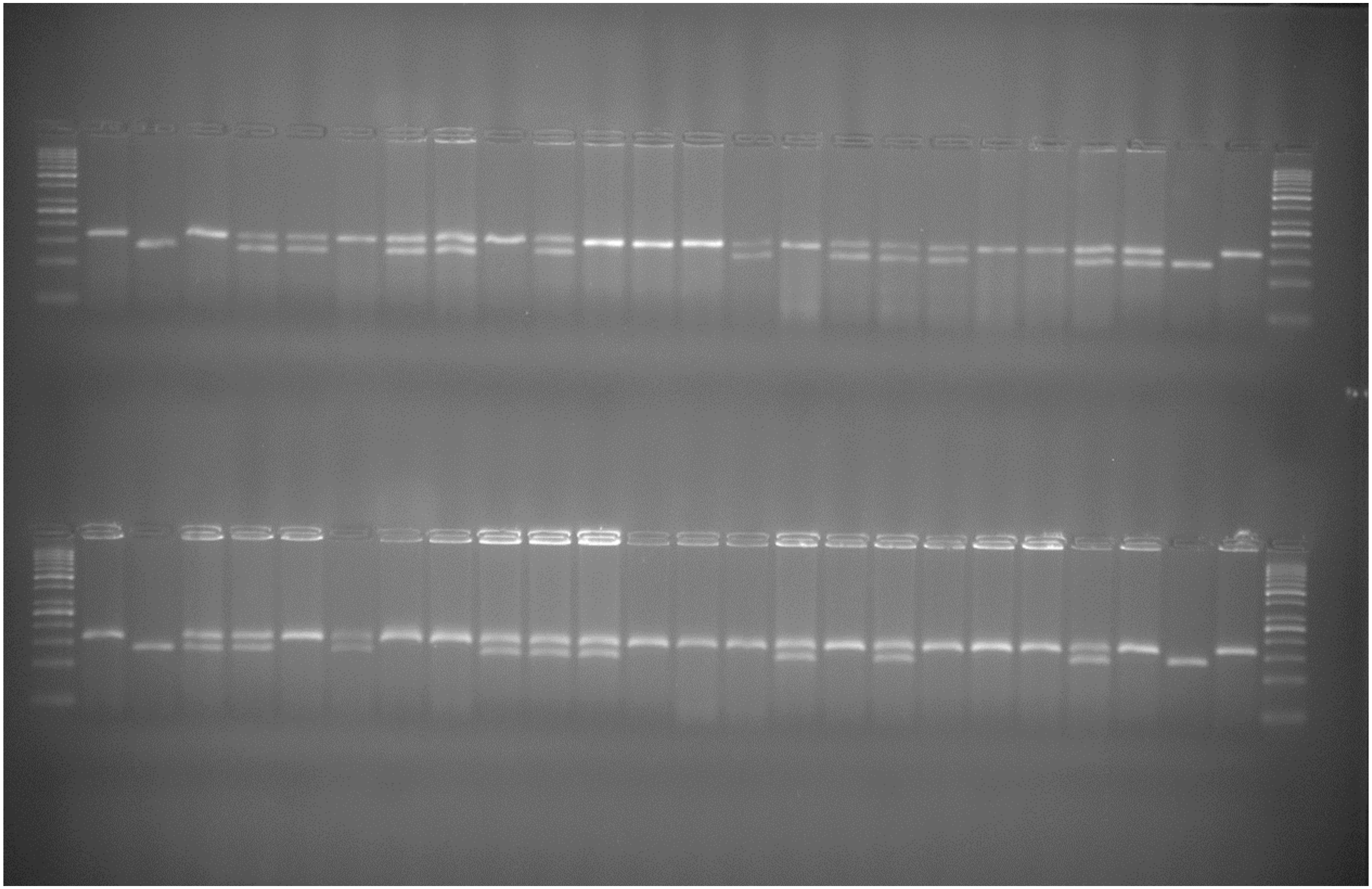

Fig 2S: Original gel image for foreground selection of o2 gene as given in Fig 2.

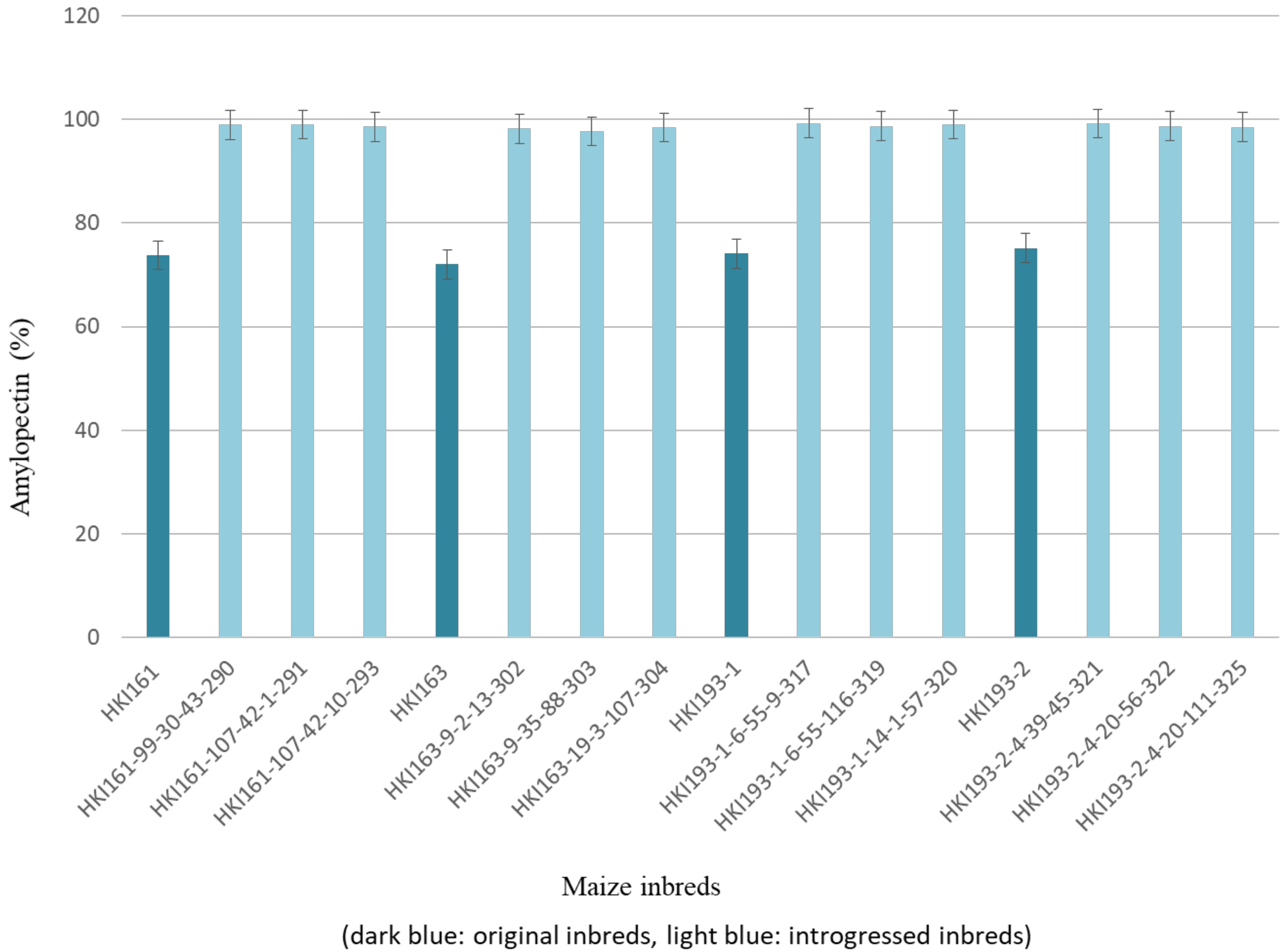

Fig. 3S. Amylopectin levels in original- and introgressed inbreds

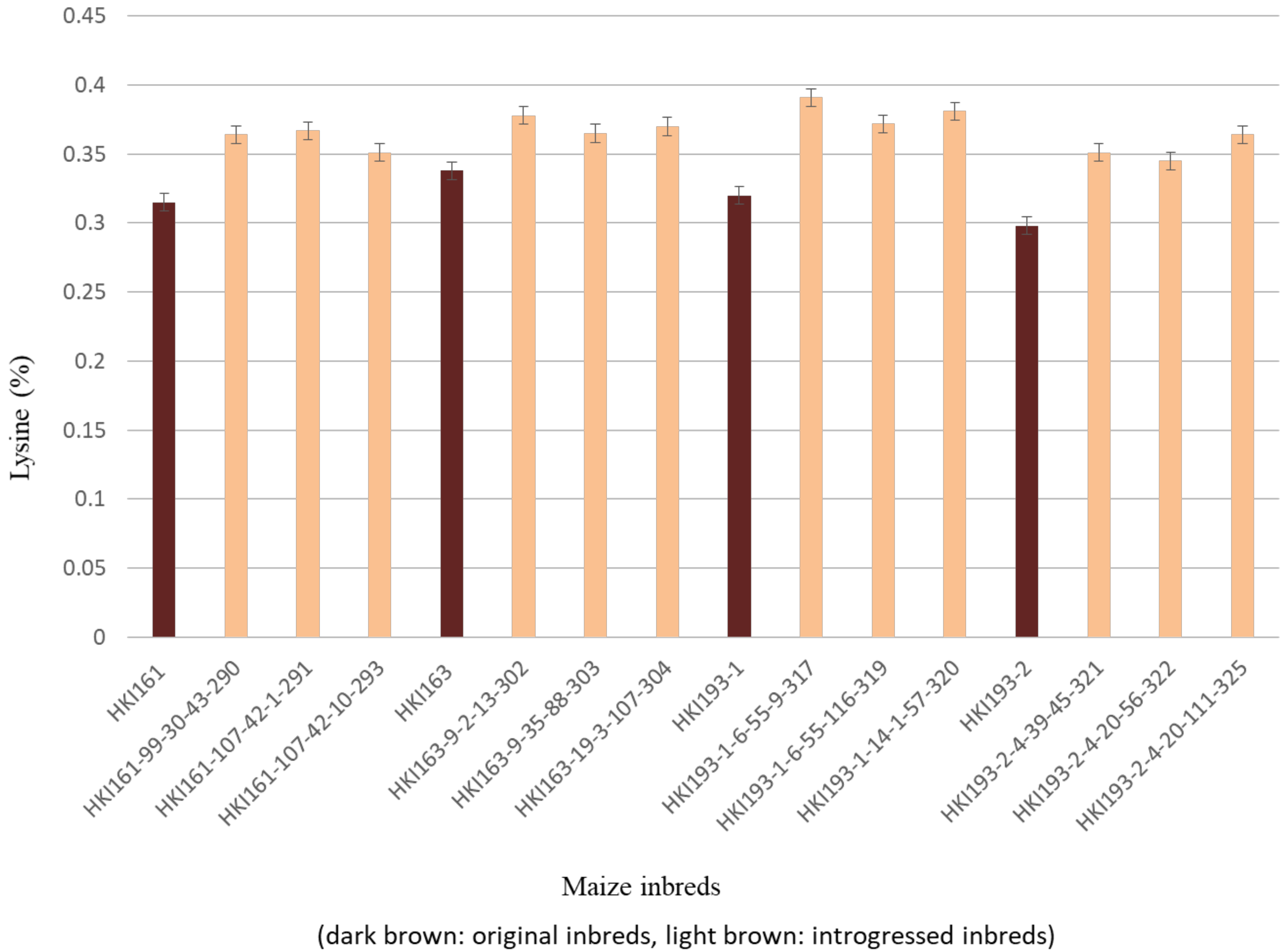

Fig. 4S. Lysine levels in original- and introgressed inbreds

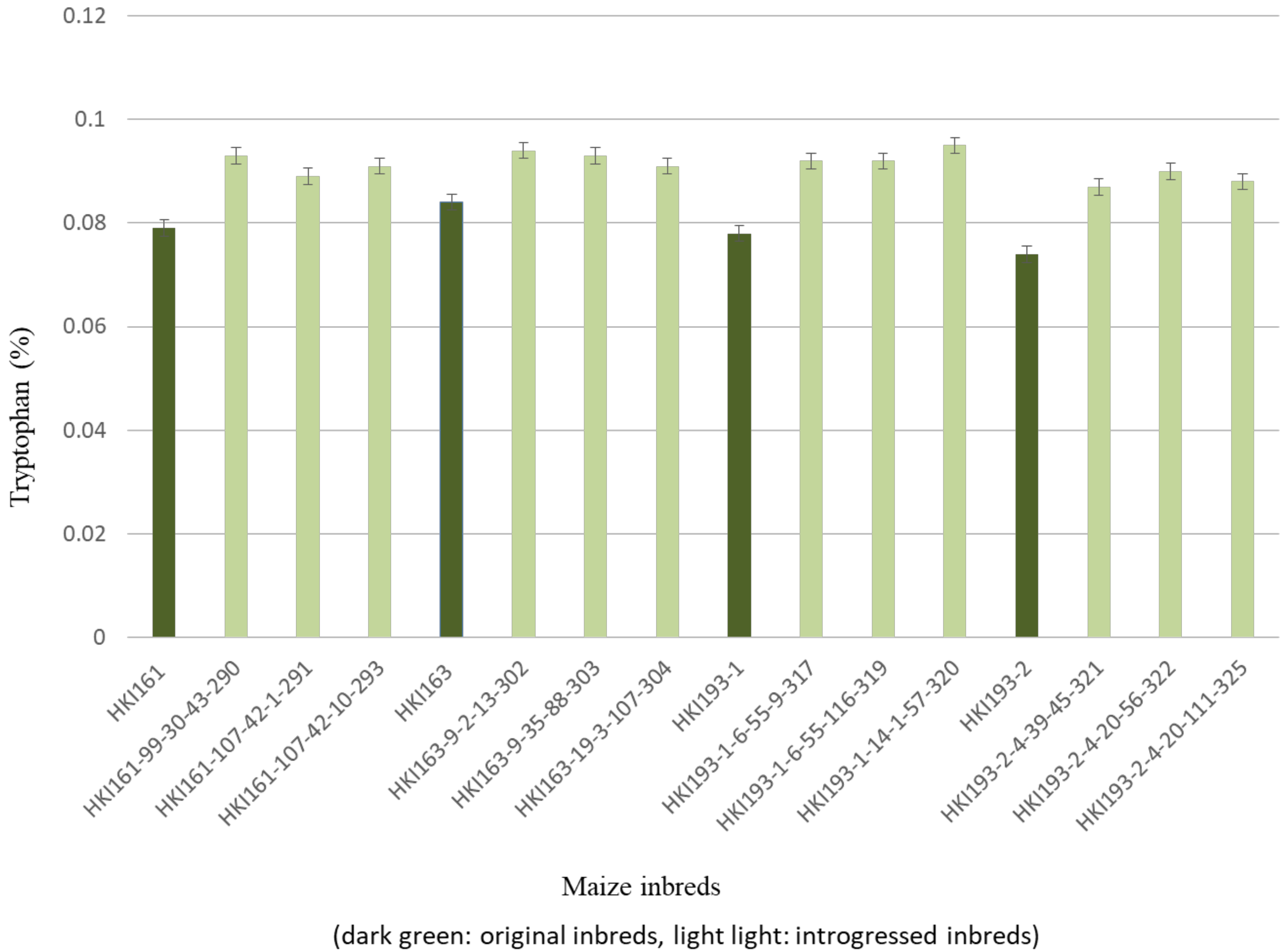

Fig. 5S. Tryptophan levels in original- and introgressed inbreds

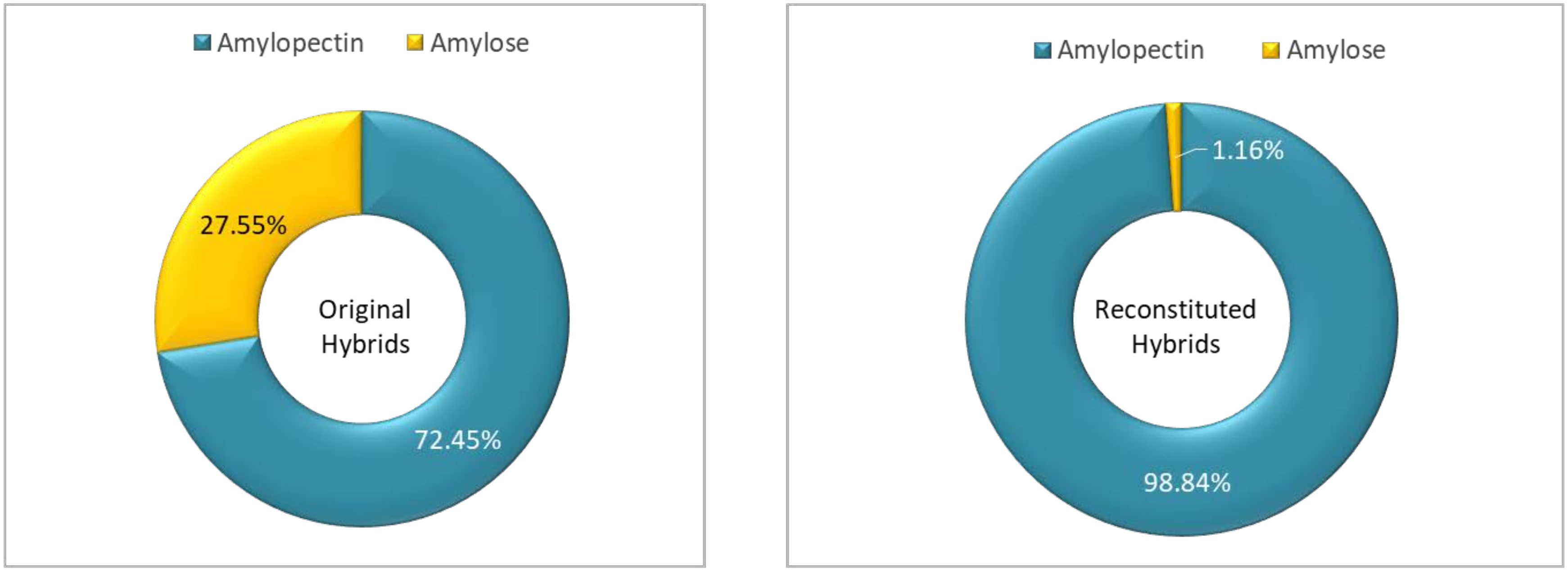

Fig. 6S. Average amylopectin and amylose proportion in original and reconstituted hybrids
